# *k*-mer approaches for biodiversity genomics

**DOI:** 10.1101/gr.279452.124

**Published:** 2025-02

**Authors:** Katharine M. Jenike, Lucía Campos-Domínguez, Marilou Boddé, José Cerca, Christina N. Hodson, Michael C. Schatz, Kamil S. Jaron

**Affiliations:** 1 Johns Hopkins University, School of Medicine, Baltimore, Maryland 21205, USA;; 2 Centre for Research in Agricultural Genomics, CRAG (CSIC-IRTA-UAB-UB), Campus UAB, Cerdanyola del Vallès, 08193 Barcelona, Spain;; 3 Tree of Life, Wellcome Sanger Institute, Wellcome Genome Campus, Hinxton, Cambridge CB10 1SA, United Kingdom;; 4 Center for Ecological and Evolutionary Synthesis, Department of Biosciences, University of Oslo, 0313 Oslo, Norway;; 5 University College London, UCL Department of Genetics, Evolution & Environment, London, WC1E 6BT, United Kingdom

## Abstract

The wide array of currently available genomes displays a wonderful diversity in size, composition, and structure and is quickly expanding thanks to several global biodiversity genomics initiatives. However, sequencing of genomes, even with the latest technologies, can still be challenging for both technical (e.g., small physical size, contaminated samples, or access to appropriate sequencing platforms) and biological reasons (e.g., germline-restricted DNA, variable ploidy levels, sex chromosomes, or very large genomes). In recent years, *k*-mer-based techniques have become popular to overcome some of these challenges. They are based on the simple process of dividing the analyzed sequences (e.g., raw reads or genomes) into a set of subsequences of length *k*, called *k*-mers, and then analyzing the frequency or sequences of those *k*-mers. Analyses based on *k*-mers allow for a rapid and intuitive assessment of complex sequencing data sets. Here, we provide a comprehensive review to the theoretical properties and practical applications of *k*-mers in biodiversity genomics with a special focus on genome modeling.

The genomics field has come a long way in the past quarter century. Sequencing and assembling even a partial genome was once a multibillion dollar accomplishment. Now, complete telomere-to-telomere (T2T) assemblies are being produced for an ever-increasing array of species (Nurk et al. 2022; Chen et al. 2023; Zhang et al. 2023), and large global efforts to capture the genomic diversity of life are well underway (Lewin et al. 2022). However, as of this writing, the vast majority of known species do not have an assembly, or even associated sequencing data. In fact, only 1% of known eukaryotes have an associated assembly according to the Genomes on a Tree tracker (Challis et al. 2023). Genome assembly is a complex process that needs to be guided by orthogonal assembly-free approaches. These methods are frequently based on a popular bioinformatic concept of “*k*-mers.” *k*-mers have proven to be an efficient and powerful concept for understanding the vast and continually growing sequencing data.

Every genomicist has encountered *k*-mers in one way or another. Although the names have changed substantially over the years (see Box 1), the concept remains the same. *k*-mers are frequently used for a representation of genomic sequences (Marchet et al. 2021), for example, in the back-end of alignment tools such as BLAST (Altschul et al. 1990), Bowtie 2 (Langmead and Salzberg 2012), and minimap2 (Li 2018) and practically all genome assemblers (for a review, see Li and Durbin 2024). More recently, *k*-mers have emerged as a popular framework for analyzing genomic data sets directly in methods such as genome profiling, thanks to the development of user-friendly tools such as GenomeScope (Vurture et al. 2017; Ranallo-Benavidez et al. 2020). Consequently, *k*-mers are now a fundamental part of genomics. To make the concept of *k*-mers more accessible to users and developers of *k*-mer-based software methods, we offer this guide. We define and explain the basic properties of *k*-mers, review the established *k*-mer-based techniques in genomics, and showcase recent creative uses of *k*-mers for complex genomic problems. Together with this paper, we provide online materials for the reader to exercise the knowledge on real biological examples available at GitHub (https://github.com/KamilSJaron/k-mer-approaches-for-biodiversity-genomics).

Box 1. **Historical perspective on *k*-mers**

The oldest reference to the concept dates back to the legendary work of Claude Shannon (1948), in which he used “**N-grams**” to develop a theory for communication, later to calculate entropy of a natural language. In the mathematically oriented research community, the concept is most often referred to as “***k*-tuples**” (Drmanac et al. 1991; Idury and Waterman 1995), including shorter versions such as “**ktup**” (Lipman and Pearson 1985), or as other prefixes such as “**L-tuple**” (Idury and Waterman 1995) or “**ℓ-tuple**” (Li and Waterman 2003). Perhaps to reach a wider audience, some authors decided to use “***k*-word**” (Lippert et al. 2002; Li and Waterman 2003) or just “**word**” (Reinert et al. 2000). Instead, it was “***k*-mer**” that became the most popular and common expression. Sequencing by hybridization used “**11-mers**” for the oligo templates (Drmanac et al. 1989), although they never used the generalized form with *k*. Although the concept of *k*-mers also appeared in the publication of BLAST as “**w-mers**” (Drmanac et al. 1989), it took nearly a decade before *k*-mer became more commonly used. The use of “*k*-mer” became more common in late 1990s, including within the pioneering work of the whole-genome aligner MUMmer in 1999 (Delcher et al. 1999). In 2000, Liu and Singh (2000) coined “*k*-mer word frequency distribution” and described it as a “signature” of the sequence (Delcher et al. 1999; Liu and Singh 2000). A few years later, Mullikin and Ning (2003) published a “word frequency graph,” which is the first record of a *k*-mer spectrum. Publications using the word *k*-mer increased in the following years compared with any other of the terms. The expression “*k*-mer” was solidified as the main way to describe this concept throughout the 2000s with the release of several genome assemblers, read aligners, and specialized software for counting *k*-mers such as Jellyfish (Mullikin and Ning 2003; Marçais and Kingsford 2011). For a few more details, see Supplemental Text S1.

## *k*-mer basics

In a genomic context, *k*-mers are substrings of nucleotides of length *k* contained within a sequence (e.g., individual reads, reference genome, or any other sequence). *k*-mers are typically used for DNA, but the concept can be applied to RNA and protein sequences as well. Any genomic sequence can be decomposed into a number of consecutive *k*-mers, and this number will depend on both the length of the sequence (L) and *k*-mer length (*k*). For example, in the following sequence: AAGTCCAT (L = 8), there are seven *k*-mers of length 2 (2-mers), six 3-mers, five 4-mers, four 5-mers, three 6-mers, and two 7-mers (Supplemental Fig. S1). The number of *k*-mers in a sequence of length L is equal to L − *k* + 1. This is a general principle that can be applied to any sequence, regardless of the sequence length or composition.

What is the point of further fragmentation of sequencing reads or genomes? At the most fundamental level, they provide a convenient way to break apart a genomic sequence into simpler words. Unlike natural language, which has the benefit of spaces and other punctuation marks, genomes do not intrinsically mark the start or end of words. Nevertheless, *k*-mers impose such a structure with surprisingly powerful outcomes. The fraction of *k*-mers perfectly matching the sequenced template will necessarily be greater than the fraction of reads that are a perfect representation in their whole entirety. For example, one sequencing error in a read means that the read is not a perfect match to the sequenced template, but only a subset of its *k*-mers will inherit the error. A read of length L contains L − *k* + 1 *k*-mers, but only *k* of them will contain the incorrect sequence (e.g., for 100 bp reads analyzed with 21-mers, a sequencing error will disrupt 21 of the 80 *k*-mers in the read, except if the error is near the end of the read). The remaining *k*-mers will represent true genomic sequence.

The second benefit is purely computational: Usually we analyze the *k*-mers that are the faithful representation of the sequenced template; therefore, we can use exact matches to count *k*-mers, which is much faster than any imperfect matching algorithm (e.g., using a hash table, or binary search instead of computing a complete alignment) and, for many applications, does not require a reference genome. Most notably, *k-*mers enable de novo genome assembly via de Bruijn graphs used in assemblers such as EULER (Pevzner et al. 2001) or SPAdes (Bankevich et al. 2012). De Bruijn graphs are constructed of *k*-mers found in the reads, followed by a series of sophisticated graph transformations, and are used in such assemblers as Verkko (Rautiainen et al. 2023) or La Jolla Assembler (LJA) (Bankevich et al. 2022), which both use a multiplex de Bruijn graph for assembling long reads (Compeau et al. 2011).

Finally, a third benefit is that *k*-mers can be used to rapidly assess various biological features. For example, they can be used to estimate genomic properties such as genome size, genome repetitiveness, and heterozygosity (Chikhi and Medvedev 2014; Vurture et al. 2017). They can also be used to estimate similarity between genomes without alignment using Simka (Benoit et al. 2016) or even subsets of representative *k*-mers in Mash (Ondov et al. 2016). Before expanding further, however, we need to understand the properties of *k*-mers and how the choice of *k* affects the data set. The direct applications of *k*-mers are the main focus of this paper with emphasis on *k*-mer spectrum and genome profiling.

### Essential properties of *k*-mers

The choice of *k* affects two essential properties of *k*-mers: the number of possible distinct *k*-mers in the set and the *k*-mer coverage. With a 4 bp alphabet, the number of possible distinct *k*-mers is 4^*k*^. However, in practice, we usually do not know the strand of the sequencing read, and as a result, the reverse complement sequences are typically counted as the same sequence; for example, CAT and ATG would be counted together. Typically, we select the lexicographically smaller of the reverse complementary *k*-mers (e.g., ATG would represent both ATG and CAT) as the sequence for the pair; the representative *k*-mers are then called “canonical *k*-mers.” Consequently, for an odd value of *k*, there are 4^*k*^/2 possible canonical *k*-mers (for more details, see Wittler 2023). Typically, odd values of *k* are used, because forward and reverse *k*-mers will be unique. In the remainder of this paper, unless stated otherwise, *k*-mers refer to canonical *k*-mers.

The size of the *k*-mer space (i.e., the number of all possible *k*-mers) increases exponentially with *k* (Supplemental Fig. S2). For *k* = 3, there are only 32 possible *k*-mers given a 4 bp alphabet; for *k* = 7, there are 8192. For these low *k* values, all possible *k*-mers will appear in a genome many times, and by chance, all these *k*-mers will likely be observed in any species. The relative frequencies of these short *k*-mers (*k* ≤ 11), sometimes called genomic signature, carry a phylogenetic signal (Karlin and Burge 1995; Fofanov et al. 2004; for a review, see de la Fuente et al. 2023). Short *k*-mer frequencies are used for alignment-free techniques for species assignment as well as detection of horizontal gene transfer candidates (for review, see Ren et al. 2018).

For most applications we need to select a *k* long enough so that most *k*-mers in the genome will be found only once. The proportion of *k*-mers corresponding to a unique position in the genome increases with a greater *k* (see Fig. 1 of Kurtz et al. 2008, or Schatz et al. 2010). For humans, the smallest *k* with some *k*-mers unique to a specific genomic location is 11, but the vast majority of the genome will be represented by repetitive 11-mers given that the human genome size is much greater than the total number of *k*-mers in the 11-mer space (Supplemental Fig. S3; Aganezov et al. 2022). We estimate the minimum length of *k* by computing the expected number of occurrences of a given *k*-mer in a monoploid genome of length G. This is estimated as G/4^k^, meaning we need to select *k* to be at least log_4_(G). For the 3 Gbp human genome, this would suggest a minimum length of 16; practically, however, the human genome has many 16 bp repeats so it is still advantageous to select even longer *k-*mers (Whiteford et al. 2005).

**Figure 1.**
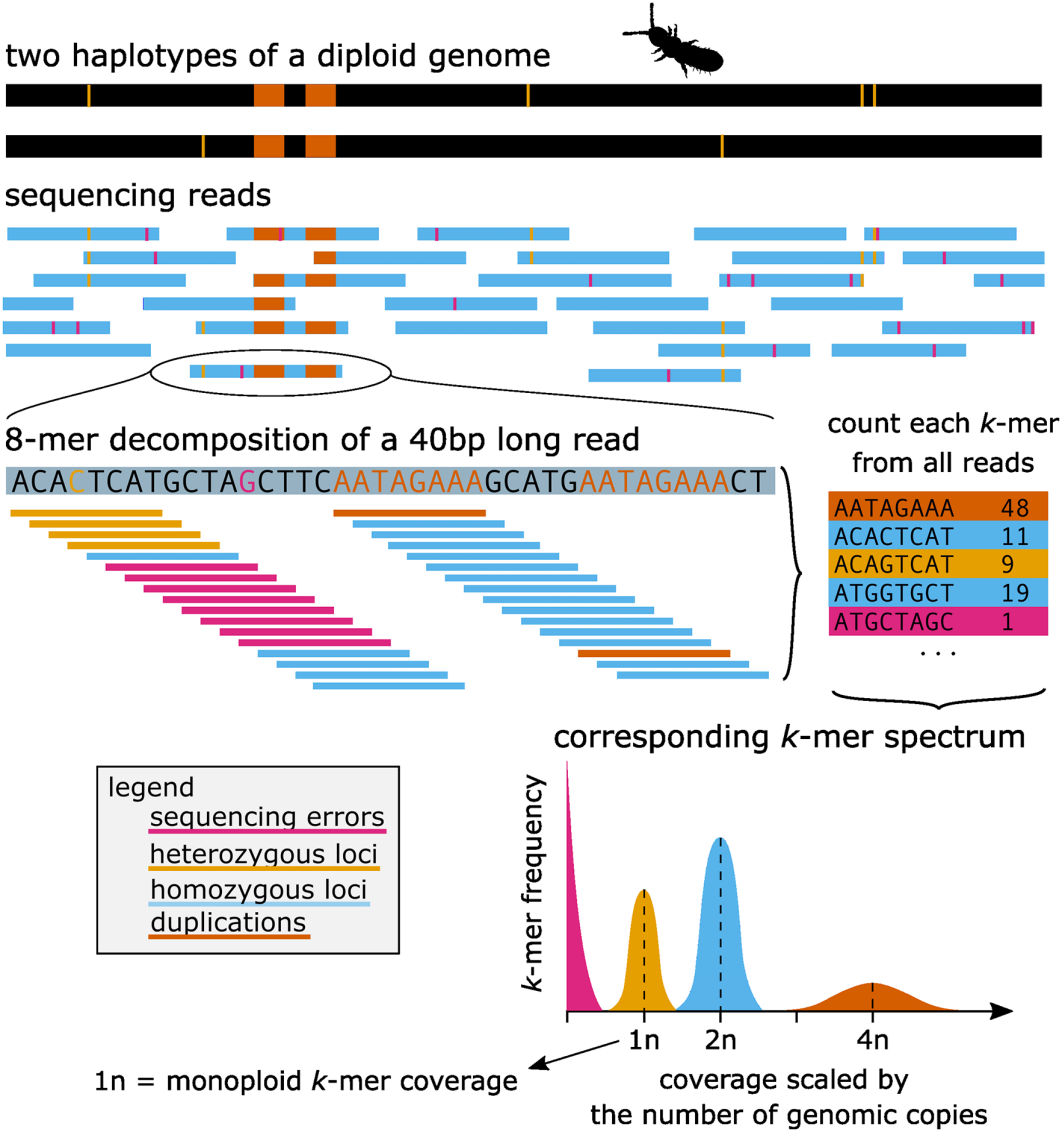
From sequencing reads to *k*-mer spectrum. This figure shows a basic illustrative example of how genomic reads can be translated into a number of *k*-mers that can be counted and represented by a *k*-mer spectrum: The example diploid genome (two haplotypes) has one duplication (orange) and six heterozygous loci (yellow). The sequencing reads contain the corresponding genomic sequence but also sequencing errors (pink). Example of a 40-base long read decomposed to *k*-mers (8-mer). Finally, *k*-mer spectra represent how many different *k*-mers (*y*-axis) show a specific coverage (*x*-axis) in the whole sequencing data set. In the vast majority of real genomic data sets, the peaks in the *k*-mer spectra would overlap, and furthermore, there would be a small number of *k*-mers representing other ploidies too (e.g., 3n for heterozygous duplications, or higher ploidies for more repetitive regions).

With *k* large enough to contain a substantial amount of *k*-mers that are unique to a single position in a genome, we can define the expected *k*-mer coverage (*C*_*k*_) as the average number of reads that contain a *k*-mer found in a single copy in the genome, also known as monoploid or 1n *k*-mer coverage, in which n denotes the expected copy number (or ploidy) of the *k*-mer in the genome. Note that the expected *k*-mer coverage (C_k_), unlike what per-base genome coverage, is sensitive to choice of *k*, read length, and sequencing error (see Supplemental Text S2).

For a given read set, lower values of *k* give a higher *k*-mer coverage, and the differences in *k*-mer coverage for different values of *k* will be more apparent for short reads (Supplemental Fig. S4). For example, using *k* = 51 for 100 bp reads generates 50 *k*-mers per read, whereas using *k* = 100 is problematic as each read is represented by a single *k*-mer only; therefore, the *k*-mer coverage in the later *k*-mer set would be substantially lower.

Importantly, each wrongly sequenced base owing to a sequencing error will propagate from reads to *k k*-mers, which will not accurately represent the sequenced template (except when the error is near the end of the read or there are multiple errors within *k* bp of each other). Therefore, the fraction of affected *k*-mers is again dependent on the chosen value of *k.* Assuming uniformly distributed errors (i.e., each sequenced position within each read has the same probability of being wrong), the probability of a *k*-mer representing the real genomic sequence is then

$$\mathrm{Pr}(\mathrm{error}\;\mathrm{free}\;k\text{-}\mathrm{mer})={\left(1-e\right)}^{k},$$

where *e* is the sequencing error rate per base. The lower *k* we chose, the greater the proportion of *k*-mers that will represent the true genome sequence (Supplemental Fig. S4) and therefore be useful for downstream analyses.

Altogether, the choice of *k* is a trade-off between proportion of the genome captured by unique *k*-mers, sequencing coverage, and error rates. Therefore, the “right” value of *k* depends on the application, sequencing depth, sequencing platform, and type of genome sequenced. For example, for short-read assembly, the choice of *k* might optimize the number of recognizable genomic *k*-mers (Chikhi and Medvedev 2014), but for high-fidelity long-read sequencing data, much higher values of *k* can be used, because the *k*-mer coverage will not decrease dramatically for higher values of *k* (see the *k*-mer coverage equation above), allowing for *k* > 1000 for certain applications. This being said, the sequencing error rates still present a limitation on the practical choice of *k*. For short-read data sets, a *k* in the range of 21 to 31 bases is typically chosen as it generates large numbers of *k*-mers from unique positions of a sequenced genome for almost any organism, while still providing high *k*-mer coverages. Furthermore, using *k* ≤ 32 is more computationally efficient than longer values of *k*, because these sequences can be represented in 64 bits, making it fast for computers to compare them (i.e., instead of comparing them character by character, they can be compared in a single computer instruction by treating the *k*-mers as 64-bit integers). Finally, for some analyses, it is also beneficial to select odd values for *k*, so that forward and reverse *k*-mers will have distinct sequences; for example, the reverse complement of AT is also AT, but the reverse complement of ACT will be AGT. The largest odd, but computationally efficient, *k* is 31, which makes it a popular choice for a wide range of applications.

## Analysis of sequencing libraries using *k*-mer spectra

One of the most common direct applications of *k*-mers in genomics is for characterization of a sequencing data set using the distribution of *k*-mer coverages; this is commonly referred to as *k*-mer spectrum or *k*-mer histogram. A typical *k*-mer spectrum of a moderately heterozygous diploid organism features four apparent coverage peaks (Fig. 1): The first peak represents sequencing errors (those with low coverage; in pink on the figure); the second peak represents unique genomic sequences from heterozygous loci (1n; yellow). This peak will be centered around *C*_*k*_ coverage, sometimes referred as “1n” or “monoploid” *k*-mer coverage. The third peak represents all homozygous loci in the genome centered around 2 * *C*_*k*_ coverage (2n; blue), and the fourth, usually a much smaller peak, represents genomic duplications centered around 4 × *C*_*k*_ coverage (4n; orange). In reality, there are frequently other coverage peaks present (e.g., 3n peak of genome duplications in heterozygous state, or sequencing of any off-target genomes such as bacterial contamination), and moreover, the peaks might overlap to the extent that a single coverage threshold might be challenging to clearly separate genomic and error *k*-mers. For these cases such separation is essential, like error correction of sequencing reads, de Brujin graph can be used to recover the true set of genomic *k*-mers (Limasset et al. 2020). However, partially blended peaks allow accurate estimation of genomic properties as well as intuitive interpretation of *k*-mer spectra.

The *k*-mer spectra analysis has become a standard step of genome sequencing (Howe et al. 2021), and the need for efficient tools was recognized by the bioinformatics community. As a result, there has been a significant improvement in performance of *k*-mer counting tools with many additional functionalities developed in *k*-mer toolkits (for an overview of popular *k*-mer counters, see Supplemental Table S1; Marçais and Kingsford 2011; Crusoe et al. 2015; Kokot et al. 2017; Mapleson et al. 2017; Mohamadi et al. 2017; Rhie et al. 2020).

Calculating the *k*-mer spectrum is just the first step. Various models can be fitted to the *k*-mer spectrum to estimate genomic features such as genome size, heterozygosity, or repetitiveness, collectively called genome modeling or genome profiling.

### Fitting a genome profiling model

One of the first genome profiling techniques was designed to estimate *k*-mer coverage and genome size (Li and Waterman 2003). The idea behind genome size estimation is practically the same as calculating *k*-mer coverage. That is because monoploid genome size (*G*) is the total number of genomic *k*-mers divided by the *k*-mer coverage (*C*_*k*_) and the ploidy level (*p*).

$$G=\;\frac{N\;(L-k+1)}{C_{k}\;p},$$

where *N* is the total number of reads in the data set and *L* is the read length, which together with *k*-mer size *k* give us the total number of *k*-mers in the data set. The total number of *k*-mers *N* (*L* − *k* + 1) can be rapidly calculated from a *k*-mer spectrum by $\sum_{c}cf_{c}$, where *f*_*c*_ are frequencies of *k*-mers with coverage *c*. Notably, the equation is robust to any type of genome repetitiveness as long as the coverage *C*_*k*_ is estimated accurately. However, this strategy for genome size estimate includes also the proportion of all the *k*-mers that represent sequencing errors, therefore artificially inflating the estimated genome size. Tools that include an error model subtract the estimated error *k*-mers, which allows for a more precise genome size estimate.

The estimation of the *k*-mer coverage (*C*_*k*_) is typically done by fitting one, or multiple, distributions to the empirically calculated *k*-mer spectra. The simplest distribution, and a natural choice for a sampling process such as genome sequencing, is the Poisson distribution (Li and Waterman 2003; Liu et al. 2013; Sarmashghi et al. 2021). A Poisson distribution is easy to fit because it is characterized by a single parameter, determining both the mean and the variance. However, in practice the observed variance is usually greater than the variance fitted by the Poisson distribution (i.e., the observed distribution is “overdispersed”) so that it might be more appropriate to fit a negative binomial distribution (Vurture et al. 2017; Becher et al. 2020; Ranallo-Benavidez et al. 2020). The latter is a generalization of the Poisson distribution and is characterized by two parameters, a mean parameter and a separate variance parameter, but has a similar shape. A normal or Gaussian distribution can be used for high-coverage values but is not appropriate for low coverage (below 10× coverage per haplotype). This is because a Gaussian distribution is symmetric around the mean, so that at low coverage it will estimate coverage values to be less than zero (negative values) that are not sensical. An alternative distribution, which particularly addresses the fact that some sites have a lower probability of being sequenced than others (e.g., heterochromatin), is the skew normal distribution such as used by findGSE (Sun et al. 2018). No distribution fits all purposes, but in general, the more complicated coverage models are better suited for high-coverage data sets, although in the case of low-coverage data, it is preferable to use the least parameterized distribution (Sarmashghi et al. 2021).

The majority of model-fitting approaches expect relatively high sequencing coverage (>10×) so that they can fit the parameters of the distributions with very few assumptions. Authors of RESPECT took a different route, designed for low-coverage (skimming) data, as well as specifically optimized for data sets in the range 0.5×–2× sequencing coverage (Sarmashghi et al. 2021). The model estimates the number of *k*-mers that are found in one, two, three, etc., counts in the genome, whereas using simple error and coverage models (Sarmashghi et al. 2021). To make the model fit possible, the authors constrained some of the parameters; for example, errors are modeled as a proportion of *k*-mers observed only once in reads, but also the individual counts of *k*-mers in the genomes are constrained by empirically observed ratios in several hundred publicly available genomes (Sarmashghi et al. 2021). These empirical constraints were generated using haploid representation of diploid genomes; therefore, we would expect reliable estimates only for haploid species or diploid genomes with low heterozygosity.

Interestingly, the *k*-mer spectrum can also reveal other properties of the genome. The genome-wide level of heterozygosity can be determined through an analysis of the number of *k*-mers in the 1n versus 2n peaks: At low rates of heterozygosity, the 1n peak will be relatively small because most *k*-mers will be homozygous, but the 1n peak grows taller with higher numbers of heterozygous *k*-mers. Only a relatively modest rate of heterozygosity is needed to elevate the 1n peak to match the 2n peak because each heterozygous variant will cause 2 × *k* heterozygous *k*-mers (assuming the variants are spaced out appropriately). In this scenario, with *k* = 21, a heterozygosity rate of 1.19% is sufficient for the 1n peak to be as tall as the 2n peak.

Three genome profiling approaches also include estimates of genome-wide heterozygosity. First, in the genomes with low overall heterozygosity (<<0.5%), heterozygous sites will generally be more than *k* nucleotides away from any other heterozygous site. Consequently, each heterozygous site will generate 2 * *k* heterozygous *k*-mers, which has been proposed as a straightforward estimate of heterozygosity (Liu et al. 2013). However, many species, including outbreeding species and hybrids, display substantially higher levels of heterozygosity (Romiguier et al. 2014; Mackintosh et al. 2019). The problem of linked variants (i.e., multiple heterozygous variants less than *k* bp apart) is addressed in GenomeScope. With the assumption that heterozygous loci and duplications are independent and uniformly distributed across the genome, the fractions of heterozygous and homozygous *k*-mers are calculated as multiplication of per-nucleotide heterozygosity estimate (for a detailed explanation with illustrations, see the supplemental materials of Vurture et al. 2017). The third approach, Tetmer, estimates the genetic diversity of a population and is based on an infinite site model and coalescent theory (Lohse et al. 2011, 2016; Becher et al. 2020). For a more detailed comparison of the three methods, see Supplemental Text S3.

The most popular tool, measured by citations to date, and the tool we will use for all the examples below, is GenomeScope 2.0, which also includes support for polyploid genomes and more advanced model-fitting methods (Ranallo-Benavidez et al. 2020). We will use it to demonstrate various uses and problems with fitting genome models, but we encourage readers to also consider other genome-fitting tools, as those might be more suitable for their specific problems (for an overview, see Supplemental Table S2).

### Common signatures of *k*-mer spectra

**Figure 2.**
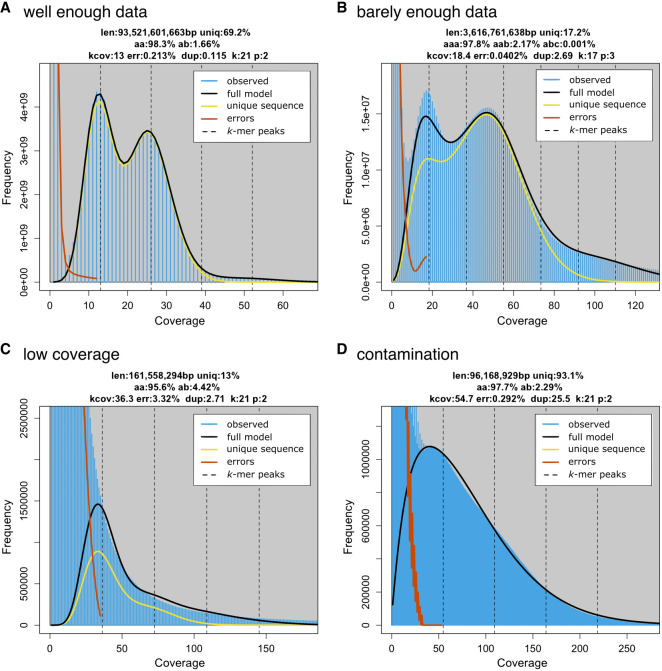
Examples of *k*-mer spectra. (*A*) *Viscum album:* a diploid spectra with enough data to observe two distinct peaks and fit a model that accurately reflects genomic features despite the large size of the genome. (*B*) *Procambarus virginalis: k*-mer spectra of a sample with low coverage, barely sufficient for a model fit. Notably, we used *k* = 17 to increase the *k*-mer coverage and make the model fit possible. (*C*) *Allium schoenoprasum*: The sequencing coverage of this data set is approximately 1×. Error *k*-mers and genome *k*-mers are completely blended; as a consequence, the model did not converge to meaningful estimates. (*D*) *Hypsibius dujardini*: a heavily contaminated sample of a tardigrade.

To generate a high-quality reference of a diploid genome, it is recommended to sequence at least 25×–30× coverage of long reads or, more generally, 15× per haplotype (Rhie et al. 2021; Darwin Tree of Life Project Consortium 2022). Even a simple visual inspection of *k*-mer spectra is valuable to quickly assess if this coverage is achieved. Such coverage should generate a *k*-mer spectrum that shows distinct coverage peaks as demonstrated by the European mistletoe *Viscum album* (Fig. 2A). Sequencing data without sufficient coverage will have poorly defined peaks, because the homozygous and heterozygous genomic peaks will be blended at the left side of the coverage plot. If the peaks are still visible, it might be possible to fit a meaningful genome model, like in the case of the crayfish *Procambarus virginalis* (Fig. 2B; see data from Gutekunst et al. 2018).

Genome profiling techniques have complicated underlying models with no known analytical solutions. They use a heuristic instead, for example, GenomeScope uses nonlinear least squares, which starts from an initial naive estimate and performs an iterative procedure to update the values until convergence. To evaluate whether a model converged well, we frequently use already known information about the species we sequence, in particular ploidy or genome size that was previously assessed via cytogenetic techniques. Confronting prior knowledge with the estimates derived from the *k*-mer spectra is often helpful in identifying potential problems in the data. In the case of the crayfish, there is a nearly perfect match of genome size estimate from the *k*-mer spectra and flow cytometry (Gutekunst et al. 2018), supporting that the model converged well. Very low sequencing coverage or elevated rates of errors lead to blending of peaks; genomic *k*-mers become indistinguishable from error *k*-mers. This is visible in the chive *Allium schoenoprasum* (Fig. 2C), in which the model (black line) does not fit the data (blue histogram) well. In such cases, the estimated values are artifacts of a poor convergence. The predicted genome size is much lower than what we would expect in the *Allium* genus, in which other species have genomes ranging from 8.4–13.4 Gbp (Henniges et al. 2023), and the coverage is much higher than what we would expect from a spectrum of this shape. Coverage problems are usually resolvable with additional sequencing, whereas high error rates may require a different sequencing technology and/or library preparation.

Contamination is another common cause of blended peaks in a *k*-mer spectrum, especially for small species that are difficult to culture in sterile environments or impossible to culture at all (Cornet and Baurain 2022). In these cases, additional species other than the target are sequenced along with the target sample, resulting in a contaminated genome profile. For example, the true genomic peak in the tardigrade *Hypsibius dujardini* is overlaid with several other cobiont genomes (Fig. 2D; see data from Koutsovoulos et al. 2016). If a contamination consists of a very few species only and the coverage is very high, we might be able to see individual peaks corresponding to the individual sources. Those are, however, usually unevenly spaced (Supplemental Fig. S5). Similar to low-coverage data sets, the values reported by the genome model are most likely inaccurate.

Different sequencing data sets show different variation in sequencing depth, but the general rule is that higher coverage leads to better separation of peaks. Sometimes, we observe blending of peaks, although the coverage is relatively high. This can be caused by various biological or technical reasons. For example, many bony fish sequencing runs show a *k*-mer spectra with a “bridge” blending the error *k*-mers and the 1n peak (Supplemental Fig. S6). This pattern has been attributed to a high proportion of tandem repeats in many fish lineages (Mackintosh et al. 2019; Reinar et al. 2023) and a coverage dropout of low-complexity A/G rich regions of Pacific Biosciences (PacBio) HiFi sequencing (Nurk et al. 2020). Another example is the well-known sequencing depth bias of Illumina short-read sequencing suffers in regard to GC content (Dohm et al. 2008; Nurk et al. 2020). This bias varies between chemistries (Stoler and Nekrutenko 2021) and is reduced with PCR-free strategies (Aird et al. 2011).

The biases above are well described and replicable when using similar samples and sequencing technologies. However, sequencing runs are also affected by sample handling, used preservatives, or occasional manufacturing problems of sequencing flow cells. Even these problems are sometimes visible in *k*-mer spectra. For example, the *k*-mer-based genome size estimate of a cape honeybee sample (142 Mbp) (Supplemental Fig. S7) is much smaller than the published genome size (236 Mbp). This was a case only for one of three samples; the other two samples from the same project (and same population) showed expected genome size. The spectra of the peculiar sample also show much higher blending of peaks, all together indicating this coverage dropout is not driven by biology of the sequenced specimen but rather technical difficulties.

In general, the lack of clearly defined and evenly spaced peaks indicates that there is no single source with sufficient coverage to generate the expected spectra. By keeping in mind the organismal biology of the target species, many of the patterns and potential problems can be anticipated.

### Common pitfalls when fitting models

The quality of fit for genome models is largely dependent on the quality and coverage of the data but also on the biological features of the genome. The most common problem of genome models is for the monoploid (1n) *k*-mer coverage to converge on a wrong value. This can happen if the 1n coverage peak is not sufficiently distinct or is significantly higher than the diploid peak. This can be caused by extremely low heterozygosity of the genome (i.e., the 1n signal is very weak) (Supplemental Fig. S8A) or by very low coverage and the 1n peak largely overlapping with the error peak (Fig. 2C).

Regardless of the genome profiling method used, when the 1n coverage is not fitted correctly, none of the estimated values will carry any biological information regarding the genome. Therefore, it is important to inspect fits and make sure the estimates agree with the context of the other known biology. For example, if we sequence a species with low expected heterozygosity (such as diploid selfing plant) and the estimated heterozygosity is >5%, it is extremely likely that the true 1n coverage is about one-half of the estimated one (Supplemental Fig. S8B). This convergence issue affects tools that expect certain levels of heterozygosity, such as GenomeScope. This is not the case for RESPECT, which assumes low or no heterozygosity. Genome profiling models are extremely useful tools, but they require knowledge of assumptions of the fitting algorithm for correct interpretation of the estimates.

Another common issue is that many *k*-mer counting tools stop counting *k*-mer coverages at a user-specified value, with a typical default of 10,000. The last value in these *k*-mer histograms typically represents how many *k*-mers have coverage greater than or equal to the highest counted coverage. This will lose resolution of the frequency of the most common repeats in the genome. For many genomes, it makes little difference; however, some extremely repetitive genomes will have a severely underestimated genome size. When using the default value, the estimated genome size of marbled crayfish is nearly half of the real genome size (Supplemental Fig. S9). When one observes unexpectedly low genome size, it is frequently caused by the truncated *k*-mer spectra. Notably, FastK does not explicitly calculate coverage of every high-coverage *k*-mer; instead, it reports as the last value of the theoretical number of *k*-mers that would span the same total sum of coverage yield as all higher coverage *k*-mers in the genome, ensuring a more accurate genome size estimate (Compeau et al. 2011; https://github.com/thegenemyers/FASTK).

### Joint interpretation of the *k*-mer spectra and the assembled genome

Postassembly, estimated genome qualities from *k*-mer models can be used to assess assembly accuracy. One simple yet informative metric is to compare the estimated genome size to the assembly size. This simple comparison is quite powerful because it can quickly indicate misassembly, especially partial duplications caused by heterozygosity. *Ilex aquifolium* (common holly), sequenced by The Darwin Tree of Life Project (Christenhusz et al. 2024) can serve as an example of mismatch between estimated genome size and assembly size. The estimated genome size of *I. aquifolium* based on *k*-mer histogram from PacBio reads is 815.6 Mbp. However, the primary assembly is marginally larger (830.1 Mbp) (Supplemental Fig. S10A), and furthermore, there are 7.2% duplicated BUSCOs in this genome, altogether indicating that there could be some uncollapsed regions in this assembly with sequences from both haplotypes inflating the gene count and (haploid) genome size. It was indeed the case for this genome, and it was resolved by purging the uncollapsed haplotypes using purge_dups (Guan et al. 2020; Christenhusz et al. 2024).

Several tools exist now to evaluate genome assembly accuracy using preassembly *k-*mers. We will specifically discuss Merqury (Rhie et al. 2020); however, this is not the only tool (Mapleson et al. 2017; Mikheenko et al. 2018; Cheng et al. 2021). Essentially, these tools compare the *k*-mers from sequencing reads to the *k*-mers present in the finished genome assembly to determine how complete the assembly is. This operates under the assumption that the finished assembly should contain most of the *k*-mers that were present in the sequencing reads, excluding low-coverage *k*-mers that are likely owing to sequencing errors, and half of the heterozygous alleles in diploid genomes if we are working with a diploid collapsed assembly (for Merqury plot example on the common holly genome, see Supplemental Fig. S10B). Additionally, Merqury estimates the consensus quality value score, which assumes all *k*-mers present in the finished assembly should be present in the read set. It then estimates the probability that a particular base is an error, which can be reported as the commonly used Phred score (Ewing et al. 1998). This approach is especially useful for species with no reference or a poorly assembled reference. However, it does require high-accuracy reads (such as Illumina or PacBio HiFi) as a reference point, and ideally, these reference reads would be orthogonal to those used for the assembly.

## Comparison of sequencing libraries using *k*-mers

*k*-mers can also be used to identify genomic differences between two sequencing libraries. This analytical approach can be used to estimate their genetic divergence (VanWallendael and Alvarez 2022), differences of repetitive content (Liu et al. 2017; Becher et al. 2022), to identify sex chromosomes if the libraries generated from different sexes or to identify tissue or individual specific chromosomes such as B Chromosomes (Vea et al. 2021). *k*-mer chromosome identification techniques can be used on their own or with well-known techniques already in use such as methods that identify chromosomes based on coverage differences or differences in sequence composition (e.g., SNP differences) between two sequencing libraries (for review, see Palmer et al. 2019). One advantage of *k*-mer techniques over other methods is a reduced reliance on a high-quality reference genome. Therefore, *k*-mer-based techniques may be a better approach in nonmodel organisms with a fragmented or nonexistent reference genome.

**Figure 3.**
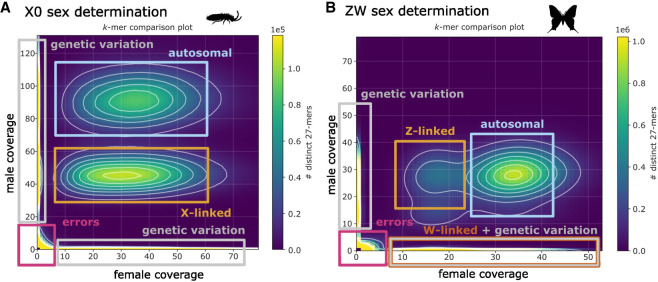
*k*-mer comparison of multiple sequencing libraries. Examples of two 2D *k*-mer spectra for species with the following: (*A*) A XO sex determination system (*Orchesella cincta*; data from Anderson et al. 2022) and (*B*) a ZW (female heterogametic) sex determination system (*Iphiclides podalirius*; data from Ebdon et al. 2024). Both plots show a heatmap of the frequency of *k*-mers in a female (*x*-axis) versus a male (*y*-axis). *k*-mers associated with autosomes or homogametic sex chromosomes (X or Z) can be differentiated in both plots (orange box) as they are at half the frequency in the heterogametic sex compared with autosomal *k*-mers (blue box). Similarly, heterogametic sex-chromosome (W Chromosome) *k*-mers can be identified in panel *B* as those that are absent in males but present in females (brown box). However, it is important to note that the autosomal genetic diversity of the two compared individuals may be mistaken for those belonging to sex chromosomes.

The basic approach in analyses using *k*-mers to identify specific chromosomes is to compare *k-*mer frequency in two sequencing libraries that differ in chromosome constitution. These 2D *k*-mer spectra, comparing *k*-mer frequency in two libraries, can be generated using KAT (Mapleson et al. 2017) and allow for the identification of *k*-mers belonging to the chromosome with a different frequency in the two samples. For instance, if identifying a sex chromosome in a species with an XO sex determination system, *k*-mers belonging to the X Chromosome will be at half the frequency in male compared with female sequencing libraries, whereas autosomal *k*-mers will be at the same frequency (Fig. 3A). The *k*-mers belonging to the X Chromosome can then be isolated and either mapped to an assembly to identify scaffolds belonging to this chromosome or mapped to reads to identify reads belonging to the X Chromosome.

Using *k*-mers to identify specific chromosomes is most effective when the region of interest is highly divergent from the other genomic regions. For instance, this approach is difficult to implement to identify the Y Chromosome in a homomorphic XY sex-chromosome system with low differentiation between the X and Y Chromosomes, as few *k*-mers will be restricted to the Y Chromosome. Therefore, situations such as this might require more permissive identification thresholds. The heterozygosity of the samples is also an important consideration. For instance, if identifying a sex chromosome from a male sample and a female sample collected from a natural population, *k*-mers covering heterozygous SNPs will show signatures of *k*-mers specific to sex chromosomes as these will also be present at half the frequency of homozygous autosomal *k*-mers (Fig. 3B). This issue can be somewhat overcome by sequencing more individuals so that individual-specific heterozygous *k*-mers can be distinguished from sex-specific *k*-mers. Finally, an important consideration is the depth of sequencing for each sample. The sequencing coverage needs to be high enough to distinguish peaks in the *k*-mer spectra belonging to chromosomes at different ploidy levels in the samples sequenced and to distinguish from *k*-mers representing sequencing errors, similar to genome profiling.

## Some creative use cases of *k*-mers

Up until this point, we have discussed general properties of *k*-mers and how to apply established *k*-mer methods to sequencing data. However, there are many problems in biology that are hard to solve with existing tools, which forces us to design novel approaches tailored to the problems and data we have. Here, we showcase the creative use of *k*-mers in three examples of such problems in three different contexts: study of germline DNA, subgenomes of allotetraploid, and species assignment.

### Extraction of germline-restricted chromosomes

A specific scenario of comparing libraries was used by Hodson et al. (2022) to identify sequences belonging to the germline-restricted chromosomes in the black-winged fungus gnat *Bradysia coprophila*, in order to explore the evolutionary origins of these unusual chromosomes. In this species, males show a peculiar set of elimination events that generate different karyotypes in different tissues and life stages (for recent review, see Gerbi 2022). Specifically, sperm carries two X Chromosomes, two germline-restricted chromosomes, and one set of autosomes, whereas the soma has a single X Chromosome, no germline-restricted chromosomes (hence the name), and two sets of autosomes. In this case, germline tissue composed mostly of sperm has a different frequency of all chromosome types compared to somatic tissue. To characterize the germline-restricted DNA of this species, approximately 95 testes of unmated males were pooled together in a single library and were sequenced separately from heads of the same individuals to generate a clean somatic-tissue library to compare with the germline.

The comparison of the two libraries using 2D *k*-mer spectra indicated there was indeed an excess of *k*-mers that occurred solely in the testes library belonging to the germline-restricted chromosomes. However, unexpectedly, the coverage of autosomal *k*-mers in the testes was still higher than the coverage of X Chromosome *k*-mers, despite the fact that autosomes are at a lower frequency in sperm (expected 2:1 ratio X Chromosome: autosomal *k*-mers in sperm). This was caused mostly by contamination of the germline library by somatic cells. A rough estimate indicated that ∼77% of the germline sequencing library was composed of somatic cells, which caused the X Chromosomes to have lower coverage than autosomes, but not one-half, as one would expect if the sequencing library was just somatic tissue (Supplemental Fig. S11). Therefore, in this case, the fact that the two tissue types had three chromosome types all at different frequencies helped to determine the relative composition of each tissue type in the sequencing libraries.

The chromosome-group specific *k*-mers were matched to contigs, which resulted in near perfect assignment of contigs to chromosomal groups (germline-restricted chromosomes, X Chromosome, autosomes). In this analysis, the quality of the assignment was particularly good for two reasons. First, because the germline-restricted chromosomes happened to be very divergent from homologous regions on the X Chromosome and autosomes (the somatic chromosomes), and second, because this gnat line had extremely low heterozygosity as it was isolated by Charles W. Metz during the 1910s (Gerbi 2022). The *k*-mer-based chromosome sorting allowed for accurate identification of tissue restricted chromosomes from a nonmodel species without a high-quality reference genome (at the time), which facilitated downstream analyses of the origin of the germline-restricted chromosomes (Hodson et al. 2022).

### Separating subgenomes of allotetraploids

Polyploid genomes are challenging to assemble owing to their large size, repetitive content that may arise from genomic shock, and the presence of chromosomes with similar composition (homeologs, or chromosomes that originated from speciation and were reunited in the same genome by allopolyploidization) (Kyriakidou et al. 2018; Wang et al. 2023). Despite these challenges, several chromosome-level assemblies of allopolyploids have been published (e.g., Yang et al. 2017; Cerca et al. 2022), in which subgenomes (i.e., the genomes that were reunited as a result of the polyploidization event) are reconstructed. In this context, *k*-mer approaches have been used to separate subgenomes, leading to significant advances in our understanding of genome evolution, including subgenome evolution, such as biases in gene retention, gene function, natural selection, and synteny (Session et al. 2016; Cerca et al. 2022; Session and Rokhsar 2023).

The separation of the two subgenomes is facilitated by the division of a lineages’ evolutionary history into three tempos (Supplemental Fig. S12): Tempo 1, the period preceding the speciation event that separated the two ancestral genomes; Tempo 2, the period between the speciation and the polyploidization events, during which ancestral genomes accumulated different transposable elements (TEs) and diverged; and Tempo 3, the period after the polyploidization event, during which both subgenomes coexist in the same nucleus. Now, consider TE accumulation in each of these separate tempos: Tempo 1, TEs accumulating in this tempo are expected to be evenly distributed on both subgenomes; Tempo 2, TEs accumulating after the speciation event and before polyploidization will be distinct on both subgenomes, as the two separate lineages will accumulate their own unique TEs; and Tempo 3, TEs that accumulate after the polyploidization event will be roughly equally distributed between subgenomes (Session et al. 2016; Cerca et al. 2022; Session and Rokhsar 2023).

In this context, *k*-mers serve as a powerful tool in subgenome separation in a chromosome-level assembly. Specifically, because evolutionary signals tend to overwrite each other on the genome, the fragmentation of the sequences offers an effective way to disentangle past processes, which have occurred in the three different tempos (Supplemental Fig. S12; Session et al. 2016; Cerca et al. 2022; Session and Rokhsar 2023). The separation of subgenomes involves two steps: first, the homeologs need to be identified. This can be done using UCEs/COS (ultra conserved elements, conserved ortholog sequence) or alignment-based approaches and synteny. When homeologs are known, *k*-mers allow identifying the accumulation of TEs in the three tempos. Specifically, by performing a *k*-mer spectrum analysis and searching for two signatures, first, we select for *k*-mers that are present in high numbers (i.e., >100×), which should come from repeated areas on the genome, such as TEs; and second, we select differently represented *k*-mers in members of the homeologs (i.e., *k*-mers which are found more than twice as often in one member compared to the other member). The assumption of high numbers targets TEs, whereas the second fishes out differentially represented TEs, thereby effectively focusing on the second tempo, when the ancestral genomes were separated and accumulating their own TEs. By doing a hierarchical clustering based on this *k*-mer separation, the chromosomes group on subgenomes.

### Species assignment using short *k*-mers

For specific types of analyses, it can be beneficial to use very short *k*-mers (*k* < 10). For the example discussed here (Boddé et al. 2022), targeted amplicon sequencing was used to analyze haplotypes averaging only 160 bp. The aim of the analysis is to identify the species by comparing the query sequences to a reference panel. As such, *k*-mers from the reconstructed haplotypes were analyzed instead of the reads directly. Furthermore, because the haplotypes can be oriented by the primers, the analysis uses the full *k*-mer set rather than canonical *k*-mers.

The trade-off in the choice of *k* is between tolerance in sequence variation and captured detail owing to the size of the *k*-mer space. Because the analysis works with reconstructed haplotypes rather than reads, the *k*-mer coverage (C_k_) does not play a role in the trade-off. For large *k*, there is little tolerance for variation between the query and the reference, whereas for small *k*, there is a high chance that the same *k*-mer is found in multiple locations in the sequence by chance. For example, in a 149 bp sequence, five evenly spread SNPs result in no 25-mers matching the reference. Conversely, the chance that all 4-mers are unique in a sequence of the same length is incredibly small (<10^−22^). Based on these trade-offs, we selected 8-mers as a reasonable length. With a mean target length of 160 bp, the chance that all 8-mers within a haplotype are unique is 84%.

To perform species assignment, we compute the *k*-mer distance from the query haplotype to each haplotype in the reference panel. The *k*-mer distance quantifies the fraction of matching *k*-mers between query and reference . The nearest neighbor sequence is the reference haplotype that minimizes the *k*-mer distance to the query haplotype. The species label is assigned by identifying the nearest neighbors for all amplicon targets of the query sample and aggregating their contributions to the assignment.

The amplicon panel and the species assignment method were developed to perform species assignment for the entire genus of *Anopheles* mosquitoes. *k*-mers provide an objective way to compare highly diverged sequences, in which multiple sequence alignment or alignment to a single reference genome tends to introduce bias toward better-represented clades in the panel and the reference species, respectively. Moreover, *k*-mers provide a natural way to incorporate small indels in addition to SNPs, which considerably increases the power to distinguish between species when working with <10 kb sequence.

## The future of *k*-mers in genomics

As essential as words are to linguistics, *k*-mers are just as essential to bioinformatics. They are one of the most basic ways to represent a biological sequence, yet their utility cannot be overstated, as they are the fundamental data type used for a myriad of applications spanning genomics, transcriptomics, and metagenomics. Building on these successes, several research trends have emerged to make them even more efficient and effective for several important applications.

Within genomics, in addition to profiling increasingly complex biological samples, *k*-mers are now being used to power several genome assembly and analysis applications. One powerful technique has been the rise of “trio binning,” in which *k*-mers identified in unassembled reads from parental genomes are used as markers to phase the unassembled reads of their children (Koren et al. 2018). This enables genome-wide phasing and de novo assembly of the individual haplotypes in a sample, which has led to a renaissance in diploid genome assembly with improved contiguity and accuracy over prior approaches (Jarvis et al. 2022), including automating the assembly of complete T2T chromosomes and genomes (Rautiainen et al. 2023; Koren et al. 2024). Another powerful technique has been a focus on “singly unique nucleotide *k*-mers” (or SUNKs) to aid in the assembly of repetitive genomes and repetitive sequences (Sudmant et al. 2010). SUNKs can be identified within unassembled reads and serve as unique sequences to “anchor” reads within a genome assembly with high confidence. They were essential, for example, to resolve segmental duplications in human genomes by identifying localized regions of unique sequence embedded within the complex repeat arrays (Vollger et al. 2019) and were recently used to assemble and validate the centromeres within the human genome (Logsdon et al. 2024). Another clever application has been to use *k*-mers as markers for variation to power genotype-to-phenotype association studies (Voichek and Weigel 2020). This is particularly powerful to tag and analyze structural variations as these are the most difficult class of variation to study from raw reads. Moving forward, we anticipate future advances assembling and analyzing more complex genomes and pangenomes using related *k*-mer-based techniques.

Relatedly, *k*-mers also play a major role in a variety of sequence classification applications. Within metagenomics, the pioneering algorithm kraken (Wood and Salzberg 2014) exploits *k*-mers as signatures of individual species, allowing fast and robust classification of individual reads without alignment. This pushed the development of several newer methods that are further optimized for performance, accuracy, and flexibility (Wood et al. 2019; Shaw and Yu 2024; Ulrich and Renard 2024). Within transcriptomics, the pioneering algorithm sailfish (Patro et al. 2014) demonstrated accurate transcript quantification was possible without alignment by using *k*-mers as markers for individual transcripts. This work led to further developments that are orders of magnitude faster and also even more accurate (Bray et al. 2016; Patro et al. 2017; Srivastava et al. 2020). We expect many future applications for *k*-mers as markers for classification and quantification of diverse samples. We also anticipate future applications in which *k*-mers are embedded into abstract semantic representations for machine learning applications (Ji et al. 2021), analogous to how word2vec (Mikolov et al. 2013), BERT (Devlin et al. 2018), and related approaches (Zaheer et al. 2020) have emerged as cornerstones in natural language processing.

Finally, one of the most important technical developments has been the rise of sampling and sketching techniques, to reduce the computational complexity of *k*-mers (Rowe 2019). The core idea of these approaches is that, for many applications, it is not necessary to exhaustively consider every possible *k*-mer in a sequence. Instead, it is often sufficient to focus on a small representative subset for an analysis, typically representing a few percentages or less of all possible *k*-mers. Because the subset is much smaller than the full list, it is substantially faster to compare the subsets, and it requires much less memory. A MinHash is a type of sketching algorithm that uses a data structure with a vector of hashes. MinHash powers the pioneering mash (Ondov et al. 2016) algorithm to quickly estimate the similarity between pairs of genomes by comparing small lists of representative *k*-mers, enabling thousands of genomes to be compared with each other on a laptop in a few minutes. More recent methods, including Sourmash (Irber et al. 2024), SuperSampler (Rouzé et al. 2023), Dashing (Baker and Langmead 2023), Bindash (Zhao 2019), and Niqki (Agret et al. 2022), have advanced the field with more concise representations, faster processing, and more flexible APIs.

Similar to the MinHash, minimizers can be used to efficiently compare the similarity of two sequences. Minimizers were originally developed to reduce the computing requirements for genome assembly and are now used to accelerate popular aligners like minimap2 (Li 2018) by focusing the algorithm to a smaller list of potential seeds. In this case, the sketch is composed of the minimizers, which are the minimal *k*-mers in a window. The current research frontier for this work focuses on developing even more advanced schemes for selecting or combining *k*-mers together to ensure the representative subset of *k*-mers is unbiased while being robust to sequencing errors and repetitive elements (for a review, see Das and Schatz 2022). There is also ongoing work to develop highly efficient software libraries and toolkits for the fast and efficient processing of *k*-mers for all these applications, especially to reduce the memory requirements for indexing large sets of *k*-mers (Chikhi and Rizk 2013; Marchet et al. 2021) and indexing *k*-mers across large sets of genomes (Pibiri et al. 2023). Notably, in a recent preprint, Chikhi et al. (2024) were able to index all 50 petabases of the NCBI Sequence Read Archive (SRA; https://www.ncbi.nlm.nih.gov/sra) using a compressed de Bruijn graph constructed from all of the *k*-mers in all of the reads present.

Overall, *k*-mers continue to be one the central concepts of bioinformatics by continuously opening new avenues for scalable analyses of high-volume genomics data. Whenever a researcher is considering profiling a genome, comparing genomes, or analyzing another alignment, they should ask themselves if it might be accomplished using *k*-mers alone.

## Supplemental Material

Supplement 1

Supplement 2

Supplement 3
