# Supplementary material for: k-mer approaches for biodiversity genomics
Source: Genome Res. 2025 Feb;35(2):219–30. doi: 10.1101/gr.279452.124 (PMC11874746; doi:10.1101/gr.279452.124)
Supplement: Supplement 1 [file Supplemental_Table_S1.pdf]

Supplemental Table S1: Overview of selected *k*-mer counting tools and tool kits

The is not an exhaustive list of *k*-mer counting tools, but an overview of those that are either historically or currently impactful. Most of the tools have their own specialised utilities no other tools does - all of them were developed with a clear intention. For references, see Supplemental Materials.

Tool<br>*Year*	*description*	*url*	*reference*
Jellyfish<br>*2011*	*Very popular and easy to use* k*-mer counter, but substantially slower compared to more recent* k*-mer counters*	*https://github.com/gmarcais/Jellyfish*	*[7]*
Khmer<br>*2015*	*A versatile* k*-mer suite that allow* k*-mer indexing, streaming and building de Brujin graphs*	*https://github.com/dib-lab/khmer*	*[8]*
KAT<br>*2016*	k*-mer counter that is also able to compare two libraries or a library and a genome, powerful diagnostic plots of* k*-mer composition vs coverage*	*https://github.com/TGAC/KAT*	*[9]*
ntCard<br>*2017*	*Very fast,* k*-mer cardinality estimator. The k-mer histogram is a fast approximation and limited to non-repetitive portion of the genome*	*https://github.com/bcgsc/ntCard*	*[10]*
KMC3<br>*2017*	*Very fast explicit* k*-mer with the capacity to calculate any arbitrary coverages users request by parameters; Interface is a bit more complex*	*https://github.com/refresh-bio/KMC*	*[11]*
Meryl<br>*2020*	*A counter that also implements efficient operations between sets of* k*-mers, like union, intersection, subtraction. Used by Merqury.*	*https://github.com/marbl/meryl*	*[12]*
FASTK<br>*2023*	*The fastest explicit* k*-mer counter, compatible with all the existing downstream tools; using a shortcut to aggregate all repetitive* k*-mers (explicitly counting only to* 2^{16}*x coverage).*	*https://github.com/thegenemyers/FASTK*	*[13,14]*
