# Supplementary material for: k-mer approaches for biodiversity genomics
Source: Genome Res. 2025 Feb;35(2):219–30. doi: 10.1101/gr.279452.124 (PMC11874746; doi:10.1101/gr.279452.124)
Supplement: Supplement 2 [file Supplemental_Table_S2.pdf]

Supplemental Table S2: List of selected genome profiling tools.

For references, see Supplemental Materials.

Tool	*description*	*url*	*reference*
Kmerfreq	*Estimates genome size from corrected long reads.*	*https://github.com/fanagislab/kmerfreq*	*[15] [16]*
RESPECT	*Primarily for skimming data; ignores the concept of heterozygosity and is pre-trained on haploid representation of genomes*	*https://github.com/shahab-sarmashghi/RESPECT*	*[17]*
findGSE	*Modelling k-mer coverage with the most complex distribution of all methods - skew normal distributionl,*	*https://github.com/schneebergerlab/findGSE*	*[16,18]*
tetmer	*focused on polyploids; based on coalescence theory*	*https://github.com/hannesbecher/shiny-k-mers*	*[19]*
GenomeScope	*using combinatorics and probability;*	*https://genomescope.org*	*[20]*
GenomeScope 2.0	*using combinatorics and probability; enhanced model accommodates polyploids; fit to transformed k-mer spectra*	*https://genomescope.org/genomescope2.0/*	*[13]*
CovEst		*https://github.com/mhozza/covest*	*[21]*
