# Supplementary material for: k-mer approaches for biodiversity genomics
Source: Genome Res. 2025 Feb;35(2):219–30. doi: 10.1101/gr.279452.124 (PMC11874746; doi:10.1101/gr.279452.124)
Supplement: Supplement 3 [file Supplemental_Materials.pdf]

Supplemental Materials: *k*-mer approaches for biodiversity genomics

Katharine M. Jenike[1], Lucía Campos-Domínguez[2], Marilou Boddé[3], José Cerca[4], Christina N. Hodson[5], Michael C. Schatz[1], Kamil S. Jaron[3]

[1] Johns Hopkins University, School of Medicine, Baltimore MD USA

[2] Centre for Research in Agricultural Genomics, CRAG (CSIC-IRTA-UAB-UB), Campus UAB, Cerdanyola del Vallès, 08193 Barcelona, Spain

[3] Tree of Life, Wellcome Sanger Institute, Cambridge CB10 1SA, UK

[4] Centre for Ecological and Evolutionary Synthesis, Department of Biosciences, University of Oslo, Norway

[5] Department of Zoology, Biodiversity Research Centre, University of British Columbia, Vancouver, BC, V6T 1Z4, Canada

Table of content

Supplemental Figures **2**

Supplemental Fig. S1: Simple example of k-mer decomposition. 2

Supplemental Fig. S2: The total theoretical number of k-mers for each k. 3

Supplemental Fig. S3: Proportion of human genome represented by unique k-mers given k 4

Supplemental Fig. S4: The relation of the k-mer coverage and k in relation to the read length 5

Supplemental Fig. S5: Example(s) of low complexity contamination visible in a k-mer spectrum. 6

Supplemental Fig. S6: k-mer spectra of bony fish computed from PacBio HiFi reads 7

Supplemental Fig. S7: Genome proofing of two cape honey bee individuals. 8

Supplemental Fig. S8: Genome convergence pitfalls 9

Supplemental Fig. S9: Genome size estimate pitfall 10

Supplemental Fig. S10: example(s) of assembly quality assessment using the k-mer spectrum. 11

Supplemental Fig. S11: Comparison of head and testes libraries in species with germline restricted chromosomes 12

Supplemental Fig. S12: Phylogenetic representation of an allotetraploid species and the accumulation of transposable elements. 13

Supplemental Fig. S13: Sizes of peaks corresponding to heterozygosity estimates by different methods 14

Supplemental Texts **15**

Supplemental Text S1: The history of calling k-mers “k-mers” 15

Supplemental Text S2: On the definition of coverage, k-mer coverages, and their approximate relationship 17

Supplemental Text S3: Corresponding fractions of heterozygous k-mers and heterozygous nucleotides 18

Supplemental Figures

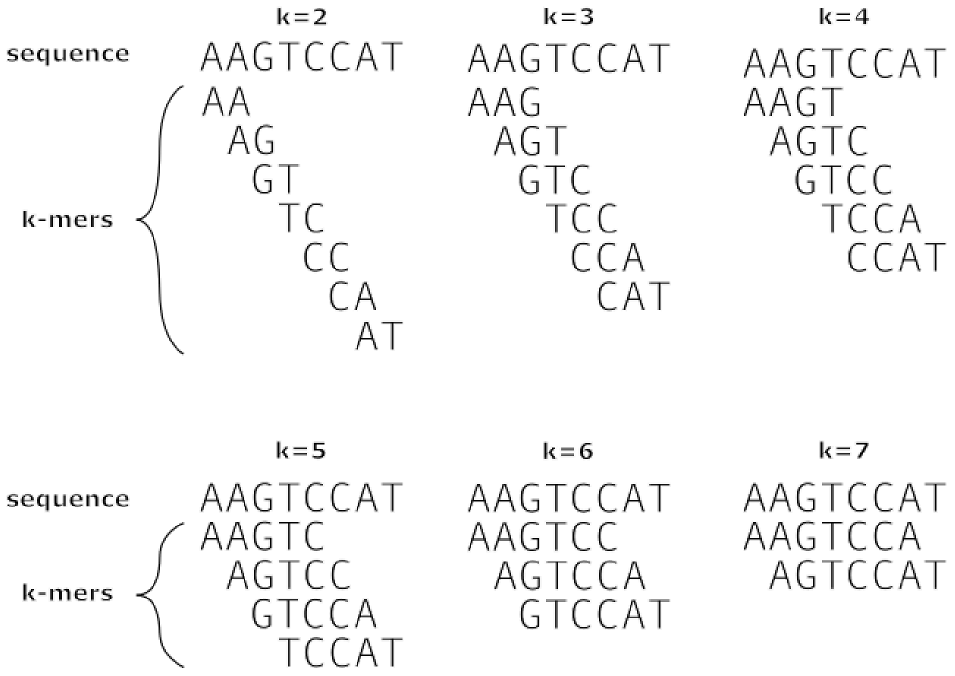

Supplemental Fig. S1: Simple example of k-mer decomposition.

The example sequence decomposed into k-mers for a range of k values, from 2 to 7. Notice the number of k-mers in a read is L - k + 1.

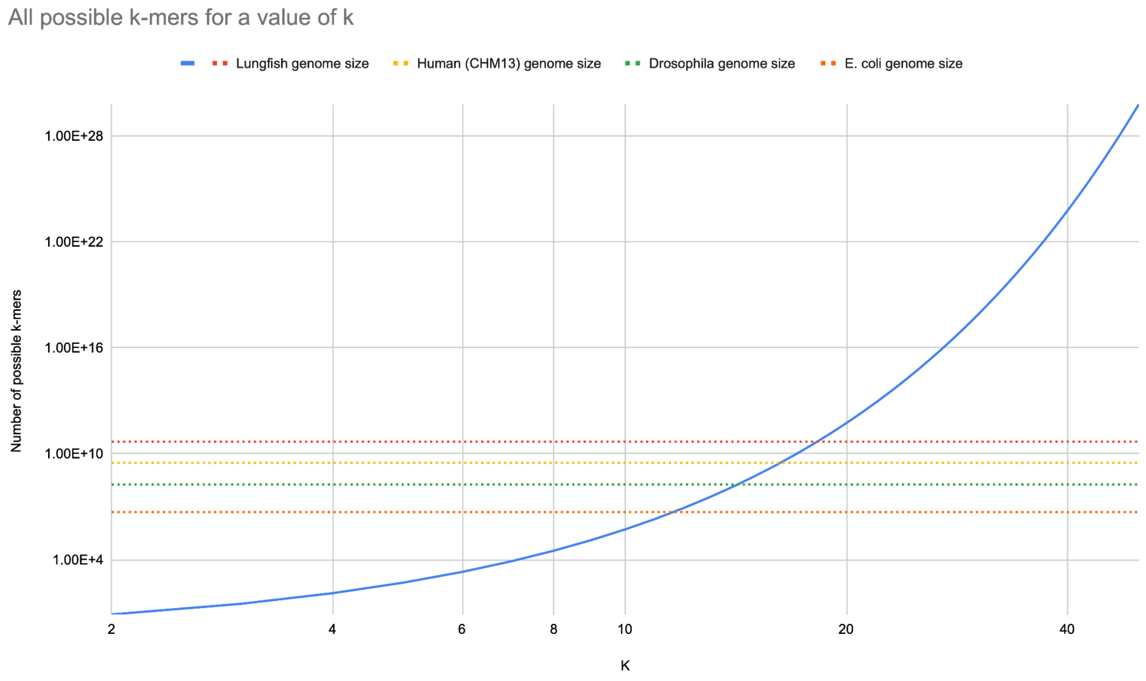

Supplemental Fig. S2: The total theoretical number of *k*-mers for each *k*.

The appreciation of the exponential increase of the complexity of the *k*-mer can be illustrated relative to genome sizes of species. The proportion of *k*-mers corresponding to unique positions in the genome will increase with *k*. Specifically, there is a guarantee at least some of the *k*-mers are non-unique if the complexity of the *k*-mer space (blue line) is below the genome size (for *k < 11*). However, with *k > 21*, the vast majority of the regions that will share the same *k*-mer sequence are most likely to be similar for biological reasons (e.g. genomic duplicates).

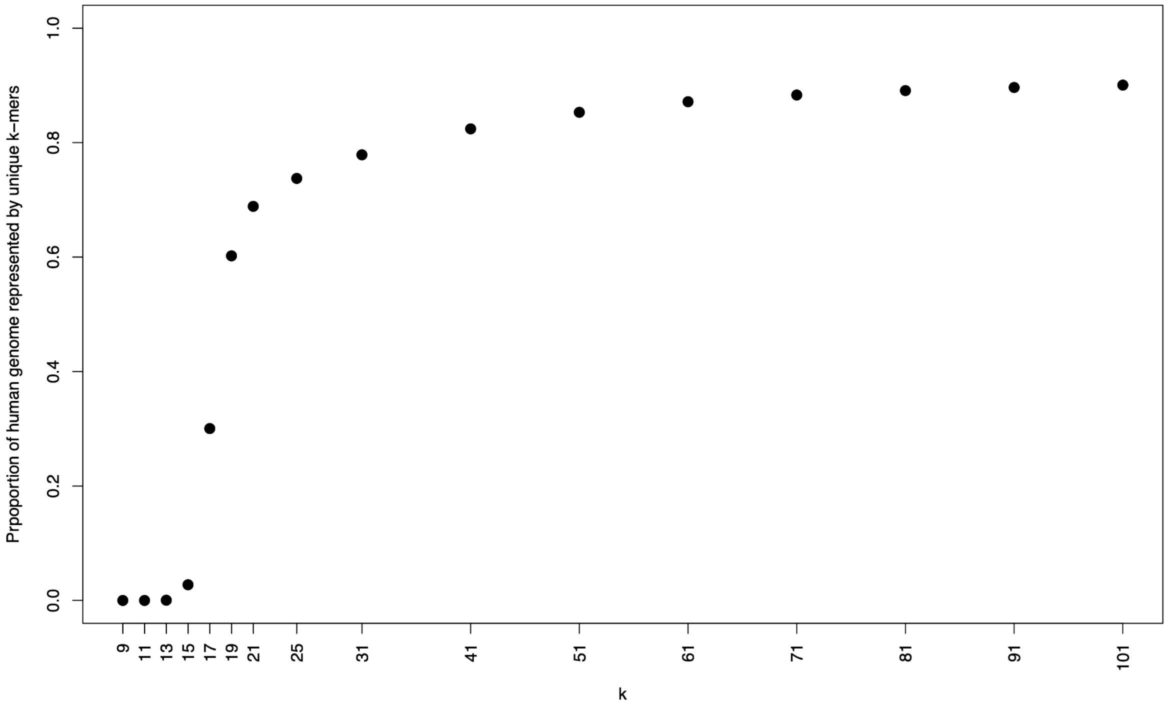

Supplemental Fig. S3: Proportion of human genome represented by unique *k*-mers given *k*

For *k* = 9 there are no *k*-mers unique to a single region in the human genome, for *k = 11*, there are 106 unique *k*-mers, which is still only a negligible proportion of all k-mers in the dataset, even though the 11-mer space contains less than $2.1x10^6$ k-mers, and the total number of *k*-mers in the human genome is approximately $3.1x10^9$. The proportion of the genome that is represented by unique *k*-mers remains very small for all values of *k =< 15*, but becomes more substantial for *k* => 17, and for k => 19 even dominant, which allows for estimating k-mer coverage and downstream genome modelling. *K*-mer values >= 31 do not substantially change this proportion which is driven mostly by longer repetitions in the genome. This plot is based on the human T2T reference T2T-CHM13v2 (Nurk et al. 2022).

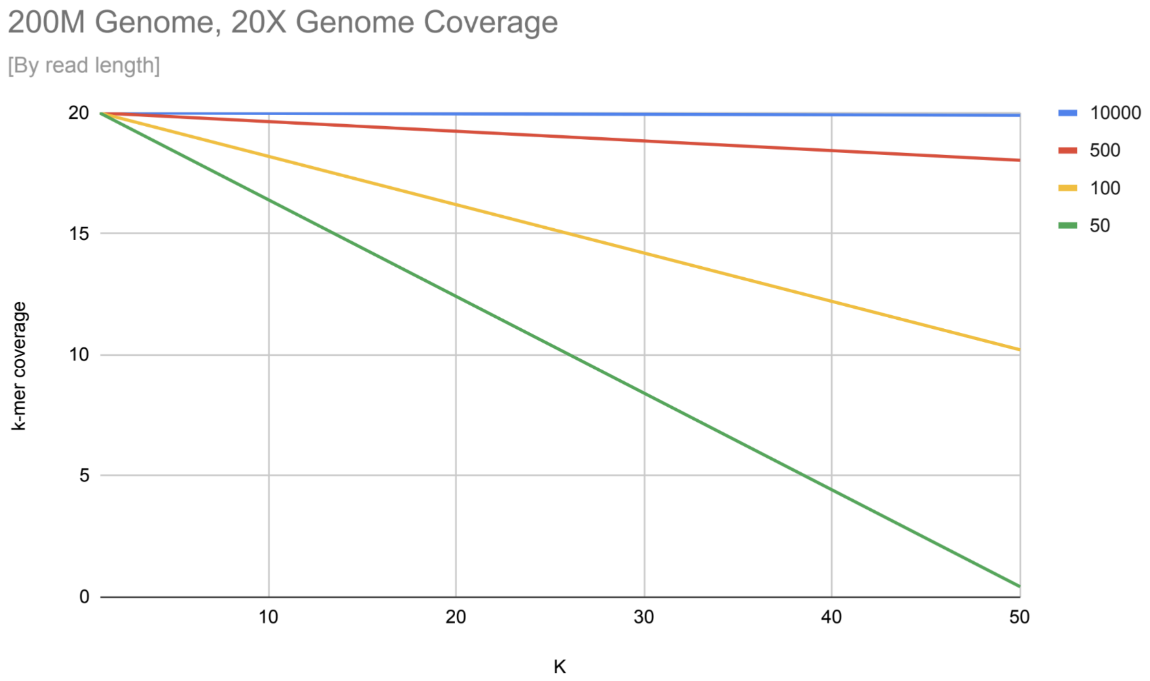

Supplemental Fig. S4: The relation of the *k*-mer coverage and *k* in relation to the read length

This plot demonstrates the *k*-mer coverage that will be observed for different values of *k* for different length reads. Each line represents a different read length, and in each case 20x average sequencing coverage is available. When *k* is low, the difference is not very prominent; for longer *k* values, the greater the benefit of long reads to maintain high *k*-mer coverage. Note that this plot does not consider sequencing errors.

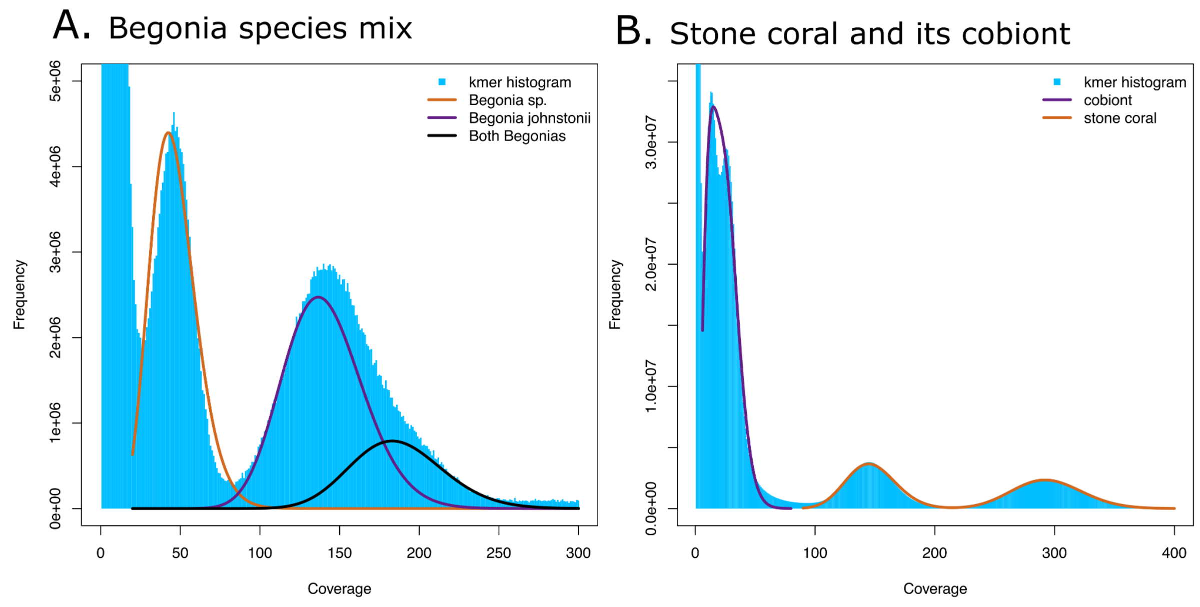

Supplemental Fig. S5: Example(s) of low complexity contamination visible in a *k*-mer spectrum.

(A) This spectrum visually shows two peaks, but they are clearly not spaced in clear stoichiometry (1:2, 1:3 or 1:4). Instead, this spectrum is the result of mixing the genomic DNA of two different plants in the same sample. The central peak (purple) represents the *k*-mers unique to the sequencing target *Begonia johnstonii*, and the lower-coverage peak (orange) represents a different begonia species that grew in the same flower pot. The black distribution represents *k*-mers shared between the two *Begonia* species. This signature is typical for sequencing genomes of two closely related species. **(B)** This spectra shows a sequencing of a mixture of two different diploid genomes. The high coverage (orange) is the target genome of stone coral *Pocillopora grandis*, while the coverage peaks (purple) represent a cobiont. Data from: https://tolqc.cog.sanger.ac.uk/asg/jellyfish/Pocillopora_grandis/

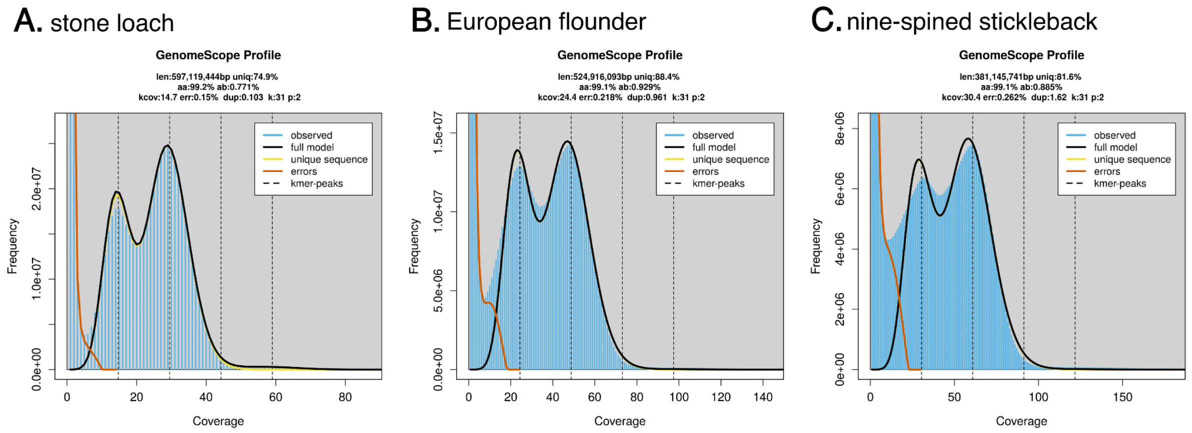

Supplemental Fig. S6: *k*-mer spectra of bony fish computed from PacBio HiFi reads

All the spectra suffer from a coverage dropout in some regions causing blending of peaks despite a relatively high coverage. While the effect is very small in the stone loach (Hänfling et al. 2023a) (**A**), it is more pronounced in the European flounder (**B**) and very apparent in the nine-spined stickleback (Hänfling et al. 2023b). This pattern has been associated with a reported coverage dropout of GA-rich low complexity regions in PacBio HiFi sequencing (Nurk et al. 2020). All three plots were retrieved from Tree of Life ToLQC portal https://tolqc.cog.sanger.ac.uk/.

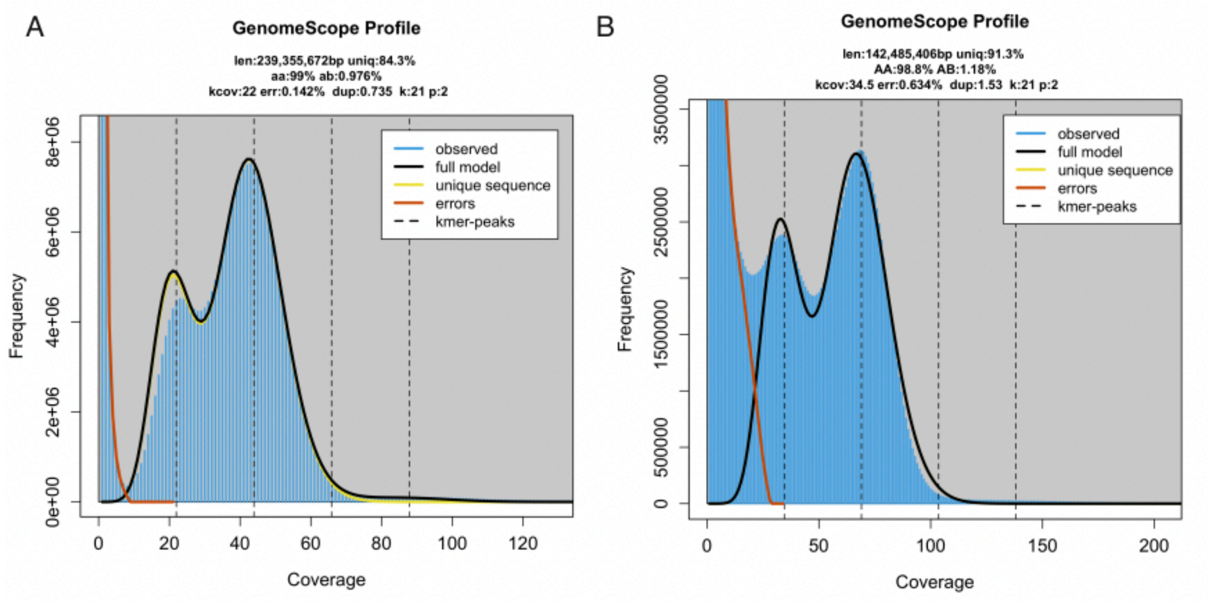

Supplemental Fig. S7: Genome proofing of two cape honey bee individuals.

Data from (Smith et al. 2019). **(A)** A *k*-mer spectra from a successful sequencing run: the error peak and genomic peaks are well separated and the predicted genome size is very close to the expected honeybee genome size. **(B)** Despite the greater *k*-mer coverage (~34x) the error peak and genomic peaks are not less separating indicating there was a problem with the sequencing library. This could potentially indicate contamination, but the genome size indicates instead there was a different problem as a large portion of the genome is missing. It is difficult to judge what exactly was wrong, but this sample is certainly not a high quality representation of a bee genome.

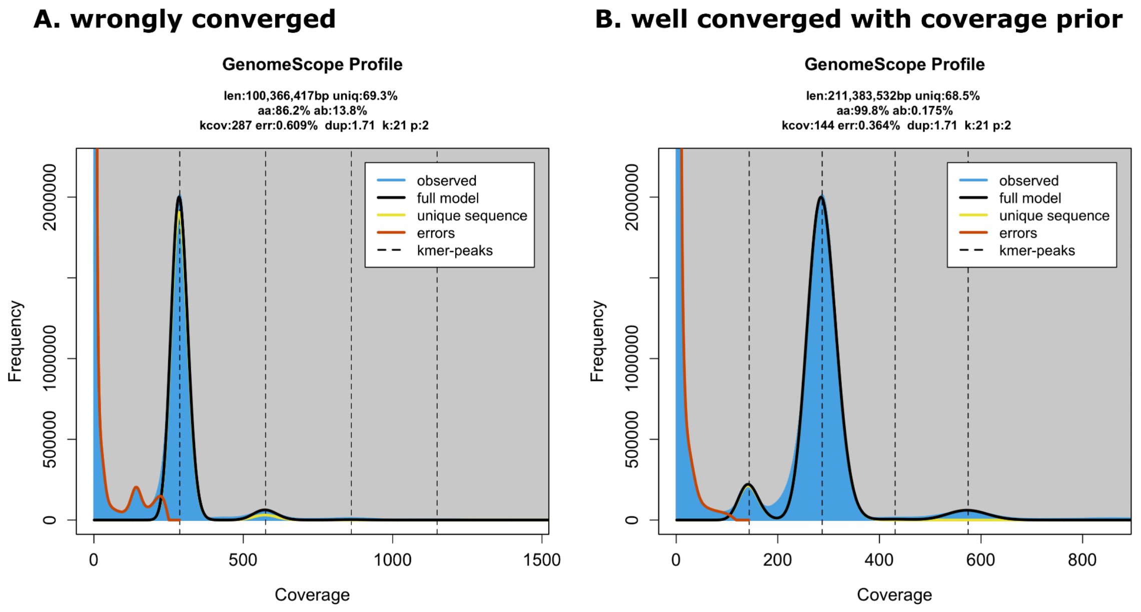

Supplemental Fig. S8: Genome convergence pitfalls

The strawberry *Fragaria iinumae* is a diploid strawberry with a typical strawberry genome size around 200Mbp. This particular specimen (DRR013884) has low heterozygosity, which is also not unexpected in a strawberry. Data from (Hirakawa et al. 2014) **A.** The default GenomeScope 2.0 run with no additional parameters misses the 1n coverage peak. One should spot several red flags: too small genome size, very high heterozygosity for a strawberry and finally, the red error line shows what could be (and really is) another genomic peak that is not part of the model **B.** GenomeScope accepts the flag “-l <prior>” which allows the user to input a (1n) coverage prior. When the analysis was rerun with specified coverage prior (-l 140), it converged on correct peaks generating biologically meaningful estimates of genome properties. On the GenomeScope web interface (https://genomescope.org) this is labelled as “Average *k*-mer coverage for polyploid genome”. Note the input value does not need to be a precise estimate, as the model fitting uses this to guide the automatic model fitting algorithm.

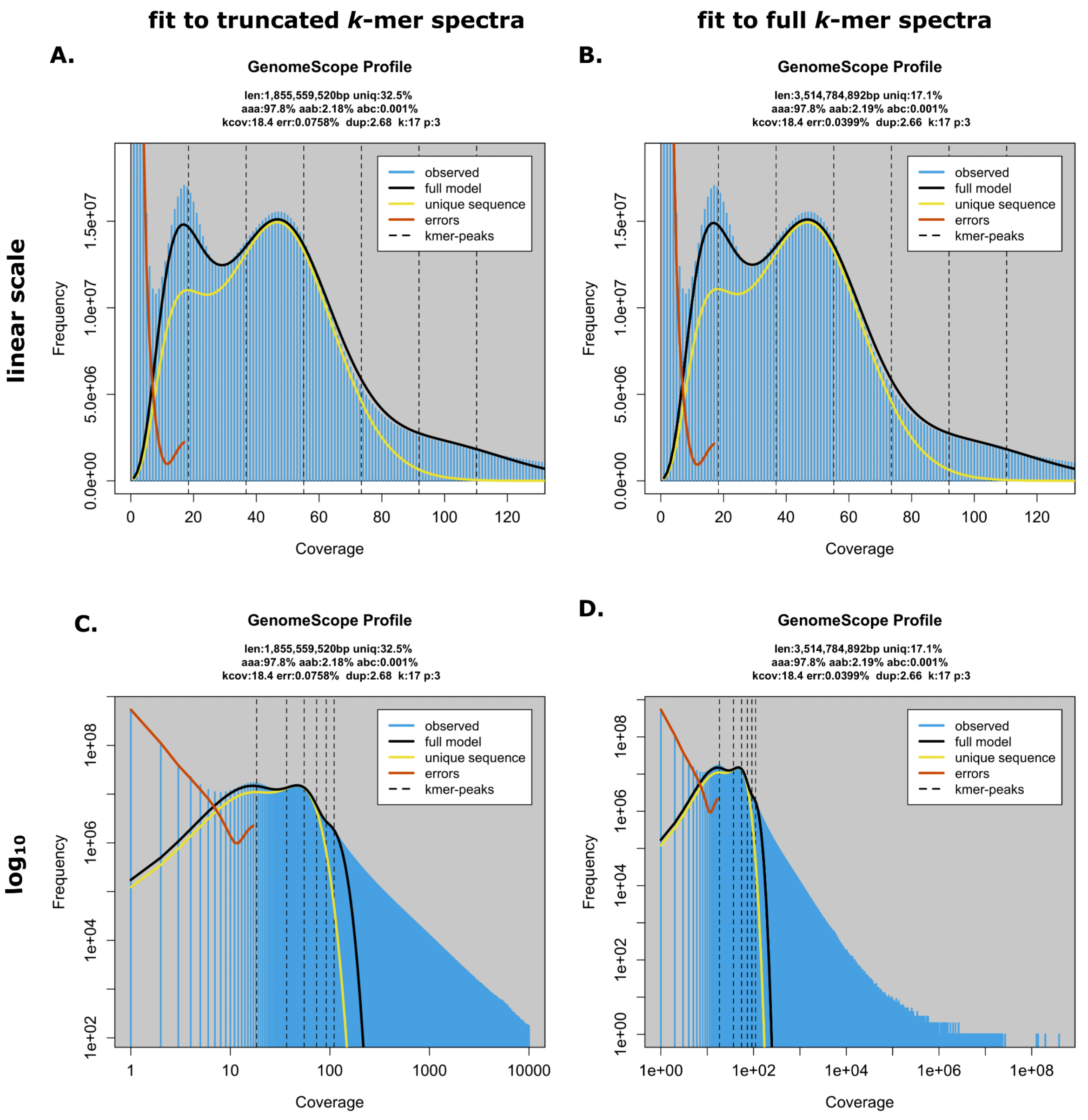

Supplemental Fig. S9: Genome size estimate pitfall

The Marbled crayfish has a triploid genome with ~3.5Gbp genome size measured by flow cytometry (Gutekunst et al. 2018). Data from (Gutekunst et al. 2018). **A.** When using the default threshold by KMC, the *k*-mer spectra is truncated at 10,000 coverage. The haploid genome size estimate is then 1.855 Gbp, which is approximately half of expected haploid size **B.** When increasing the coverage threshold to 500,000,000, the estimated genome size (3.515 Gbp) is close to the expected values given the flow cytometry measurements, indicating that a substantial proportion of the genome is on extremely repetitive sequences, which can be observed on the $\log_{10}$ scale plots (**C.** and **D.**).

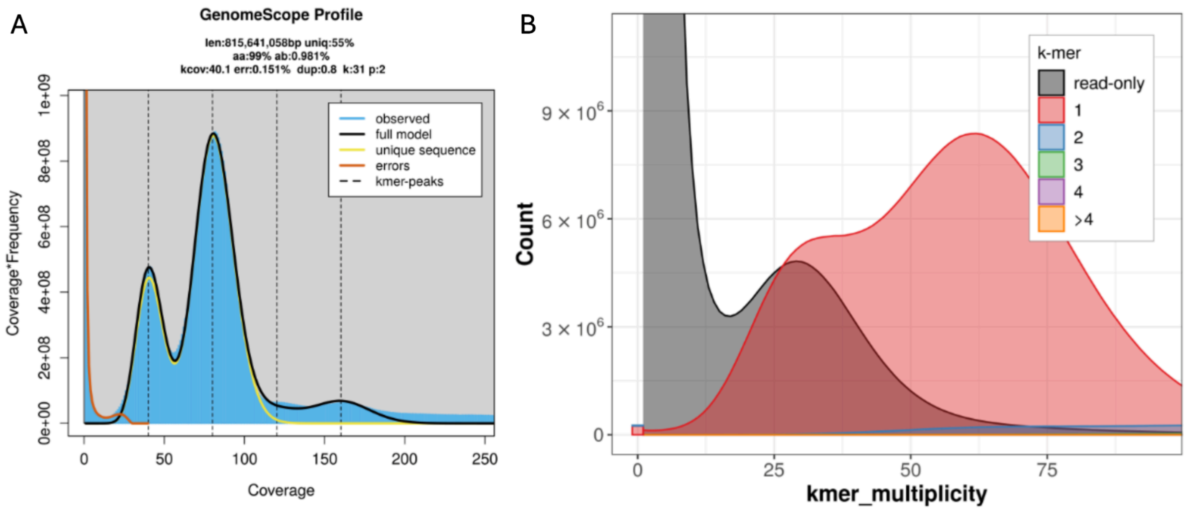

Supplemental Fig. S10: example(s) of assembly quality assessment using the *k*-mer spectrum.

(A) The PacBio HiFi 31-mer spectrum of *Ilex aquifolium* from DToL. The model fit indicates the genome is around 815.6 Mbp, with a fairly high level of heterozygosity (~1%). **(B)** Merqury plot for assembly QC of the same species. The black area represents the *k*-mers present in the read set but not in the assembly, and the red area represents what is present in the assembly once. Larger assembly size than the genome size estimate, higher 1n peak in the assembly (red) than absent (black) and relatively high BUSCO duplication scores all indicate there are uncollapsed haplotypes in the haploid genome assembly which will required downstream haplotype collapsing. Data from https://tolqc.cog.sanger.ac.uk/darwin/dicots/Ilex_aquifolium.

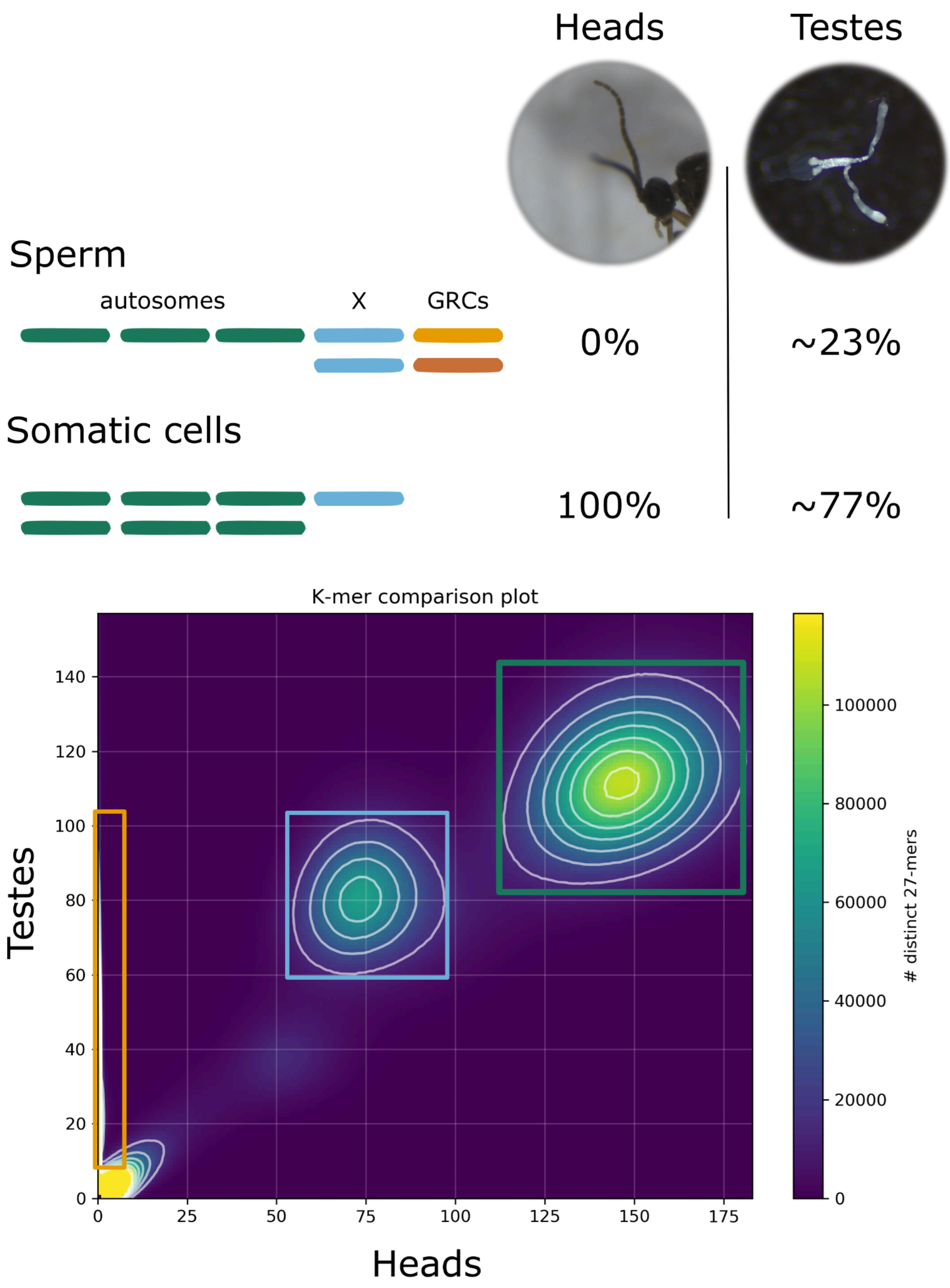

Supplemental Fig. S11: Comparison of head and testes libraries in species with germline restricted chromosomes

The two compared libraries are heads and testes. While heads are pure somatic cells with uniform karyotype, the testes are a mixture of somatic cells surrounding the germ-line and germline consisting mostly of sperm. The approximate proportion of sperm and somatic cells were calculated using mean coverages of X chromosomes and autosomes. Sperm cells contain two diverged germline restricted chromosomes (GRCs), that show in the 2D *k*-mer plot in the orange square.

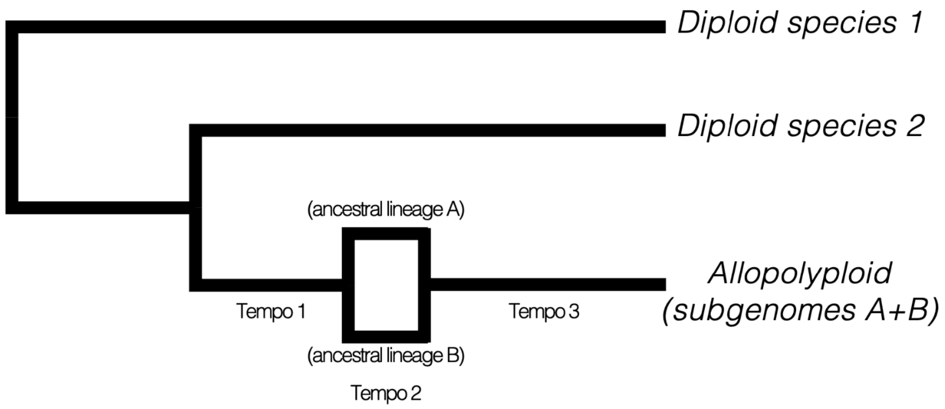

Supplemental Fig. S12: Phylogenetic representation of an allotetraploid species and the accumulation of transposable elements.

Tempo 1 occurs after the speciation event, between diploid species 2 and the allopolyploid. The accumulation of transposable elements during tempo 1 will be evenly represented across both subgenomes. Tempo 2 represents a period where lineages of the allopolyploid are segregated and accumulate differences. Transposable elements accumulated in tempo 2 will be unique to each subgenome. The third tempo begins with the polyploidization event. Transposable elements in this period will be common to both subgenomes.

Supplemental Texts

Supplemental Text S1: The history of calling *k*-mers "*k*-mers"

The concept of decomposing sequences of letters in all possible subsequences is widespread across multiple disciplines - from linguistics, through information theory to genomics and biology. Being so useful, this concept was developed independently several times under many different names. Computational linguists called the substrings -grams, mathematicians called them -tuples or -tups, in biology, the most frequent expression is -mer. Some authors recognized the difficulty in communicating the concept, so they decided to use simply -words instead, but unfortunately that led to even more confusion. In many cases, the substrings had specific length, and then authors would use concrete numbers as prefixes, e.g. 11-mer for a polymer of length 11. But sometimes, the length was just a variable, so people used various letters to mark the unknown and these letters also vary a lot.

-grams

Probably the oldest reference to the concept dates all the way back to (Burkhardt et al. 1999; Shannon 1948), Shannon used "N-grams" to develop a theory for communication, later to calculate entropy of a natural language. This is likely the oldest record of the *k*-mer concept (in the sense of all possible substrings of a certain length). The concept received a lot of appreciation in the 1990s for applications as approximate string matching, and quite often referred to as "q-gram". In 1999, a tool QUASAR was published - q-gram based database search using suffix arrays (Burkhardt et al. 1999).

-tuples

In maths, tuples are ordered sets of elements. The individual letters are the elements, but it is their order that is really important for defining each subsequence - this definition emphasises that ATGA is not the same sequence as AATG although it has the same elements. A shorter version of this notation (ktup, controversially, without a dash between of k and tup) was used in the, these days legendary, FASTA method for amino-acids sequence alignment (Lipman and Pearson 1985). There was a lot of work done on -tuples till early 2000s when they were slowly replaced by *k*-mers. Interestingly, authors working on -tuples used all sorts of prefixes: k-tuple (Idury and Waterman 1995) (Drmanac et al. 1991) REF, L-tuple (Idury and Waterman 1995) or ℓ-tuple (Li and Waterman 2003). The transition from k- to L- happened in (Idury and Waterman 1995), where they use k-tuples are a theoretical string and L-tuples are all the possible sequences of the length of a genome (which would allow perfect sequencing by hybridization, for the record, this will be one of those numbers higher than the number of the atoms in the universe for even a modest genome).

-words

This expression was introduced by a research group led by Waterman in early 2000s (Reinert et al. 2000), even specifically with the k- prefix (Mandeles 1968; Lippert et al. 2002). Perhaps one curiosity a careful reader might have noticed is that the very same research group used various forms of -tuples in the past (see the section above), so perhaps the best explained as an attempt to make the concept more accessible.

-mers

Finally, the most common term in bioinformatics these days is *k*-mer, which is simply for polymer of length k, although it is hardly ever used for anything else than a nucleotide sequence. The first record of -mer I found was from 1968 by Mandeles (Mandeles 1968).

With understanding that string of nucleotides might have a unique position in the genome - they called these oligonucleotides unique-mers. Namely, they were placing two uniqe-mers referred as Ψ-mer and Ω-mer respectively. Two decades later, sequencing by hybridization (SBH) was proposed as a new alternative to sequencing on gels; The idea was to hybridise the sequence on a chip with short nucleotide probes (5, 8, or 10 nucleotides); The only challenge was losing the positional information, which naturally created the problem of "*k*-mers" - unplaced genomic substrings.The 11 bases long nucleotide sequences were called 11-mers (Drmanac et al. 1989). Which became the standard for the following papers on the topic. One notable exception is the original description of microarrays, where they were referred to as 15 nucleotide oligomers (Chee et al. 1996), however then also used "15-mer" in the product descriptions once the commercial product (Affymetrix chips) were released. These are sequences of a specific length, not conceptually utilising the idea of taking sub-sequences of any arbitrary length. Which is of course understandable, that is a pre-sequencing era. The true *k*-mers appeared in the publication of BLAST in 1990, however using w as a prefix (w for word), so "w-mers" (Altschul et al. 1990). Nor w-mer or *k*-mer received too much attention in this era. Majority of people using this concept were coming from mathematical or computer science backgrounds and used other terms mentioned above. The use of "*k*-mer" became more common in late 1990s, including within the seminal work of MUMmer in 1999 (Delcher et al. 1999). In 2000, Liu & Singh coined "*k*-mer word frequency distribution" and described it as a "signature" of the sequence (Liu and Singh 2000). One of the other pioneers of expression "*k*-mer" were Mullikin & Ning in the Phusion Assembler publication (Mullikin and Ning 2003). In the paper they use this expression as well as plot "word frequency graph", which is one of the earliest *k*-mer spectra plots (Mullikin and Ning 2003). Publications using the word *k*-mer increased in the following years compared to any other of the terms. This gradual process was likely completed with the release of several tools including a very popular *k*-mer counter Jellyfish ((Mullikin and Ning 2003; Marçais and Kingsford 2011). This counter served for a long time as the goto *k*-mer counter and likely played a role in solidifying the expression "*k*-mer" as the main way to talk about this concept.

Supplemental Text S2: On the definition of coverage, k-mer coverages, and their approximate relationship

Historically, the coverage was a concept that was aiming to monitor progress of genome sequencing efforts by cloning (Lander and Waterman 1988). In this context, the "redundancy of coverage" was defined as

$$C = (N \times L) / G,$$

where L is read length, N is the number of reads and G is the expected genome size of a single haplotype (Lander and Waterman 1988). However, soon enough, this number became just a preliminary proxy for the expected read depth on each individual base (see (Sims et al. 2014) for a review). A better estimate of such coverage (C_g) is through investigation of the empirically mapped reads on a reference, as reported by current sequencing efforts (Sims et al. 2014; Darwin Tree of Life Project Consortium 2022). The coverage is sometimes reported as a sum of all haplotypes together and sometimes per haplotype (usually referred as 1n coverage). Notably, the original coverage is close to the empirical per-base coverage if all sequencing was of the target genome and that is regardless of the error rate as long as it does not interfere with mappability of reads. If compared to 1n sequencing coverage, the corresponding C also needs to be divided by the ploidy of the sequenced genome.

k-mer coverage is the number of times we see a *k*-mer in the readset. In theory, we could define an analogous] relationship as C was defined above but for *k*-mers, but that is not very practical for any genomic applications - sequencing runs contain many other sequences than the target genome and furthermore, we often do not know genome size in advance and finally, considering error *k*-mers as part of the coverage would imply we would need to have the ability to somehow match them to their correct contra-parts. Instead, we define the expected k-mer coverage (C_k) as the expected number of *k*-mers matching a single copy *k*-mer in the genome. Such coverage can be inferred using fits of sequencing coverage depth models to a *k*-mer frequency spectrum without knowledge of the genome size. Intuitively, it is where the first peak in the k-mer histogram is (as shown on **Figure 1** or **2**). C_k can be approximated from C_g by

$$C_k = C_g(L - k + 1) / L$$

However, this is not considering levels of contamination in the sequencing dataset, nor sequencing errors. In extreme cases those two might show widely different values. While C_g is unaffected by sequencing errors, C_k is - higher error rate there is, smaller expected *k*-mer coverage. Assuming a simple per-base error model, the fraction of genomic k-mers in the dataset is $(1 - e)^k$ which can be jointly used for a more accurate approximation of expected genomic and *k*-mer coverages.

$$C_k = C_g(L - k + 1) \times (1 - e)^k / L$$

We demonstrated this relationship in an online material: https://github.com/KamilSJaron/k-mer-approaches-for-biodiversity-genomics/wiki/demonstrating-the-effect-of-sequencing-error-rate-on-k-mer-coverage. However in practice, the

differences in C_g and C_k are frequently small enough to be unimportant for practical qualitative assessment of sequencing datasets.

Supplemental Text S3: Corresponding fractions of heterozygous *k*-mers and heterozygous nucleotides

The main text outlined the principle that heterozygous sites in a genome generate twice as many *k*-mers of half coverage than homozygous sites. Specifically, if the variant is a SNP and no other variant occurs within *k* nucleotides, there will be 2 * *k*, heterozygous *k*-mers generated: *k* *k*-mers from the maternal allele, and *k* *k*-mers from the paternal allele. This is the **simplest model** on how to model heterozygosity, but is not realistic for moderately or highly heterozygous genomes.

The **GenomeScope** model overcomes this problem by considering the probability of observing homozygous and heterozygous *k*-mers respectively given a single per nucleotide heterozygosity parameter r - the probability of observing a heterozygous nucleotide (Vurture et al. 2017). In a simplified case (modelling two peaks only) the probability of observing a completely homozygous *k*-mer is (1 - r)^*k*, which can be complemented by the probability of observing a heterozygous *k*-mer pair 1 - (1 - r)^*k* (and given two *k*-mers are generated, there will be a factor 2 in the model fit to a *k*-mer spectrum) (Vurture et al. 2017). Important here to note, that first GenomeScope fits 4 peaks also including duplications, and considers random overlap of duplications and heterozygous sites (see supplementary materials of (Vurture et al. 2017) for a very well-illustrated explanation). The GenomeScope 2.0 expanded this up to hexaploidy, and added many important features that further improved the fit, however, the fundamental logic of the fit remains the same (Ranallo-Benavidez et al. 2020). The GenomeScope model is still somewhat “unrealistic” for several reasons: different regions within genome have different probability of being heterozygous (i.e. heterozygosity is not uniformly distributed in a genome); many variants are not just SNPs; and/or a large proportion of the genome might be covered by repetitions with more than two copies. How much of a problem this presents in the estimates is still an open question, and the answer is most likely dependent on the studied species.

Finally, **Tetmer** (Becher H, Brown MR, Powell G, Metherell C, Riddiford NJ, Twyford AD 2020) is a tool that estimates genetic diversity (θ) as opposed to heterozygosity (fraction of nucleotides that differ between haplotypes). Specifically, the method assumes no *k*-mer recombines within, and the probability of a homozygous *k*-mer pair is $\theta_k / (\theta_k + 1)$, where θ_k is a per-*k*-mer genetic diversity. It is suggested for θ_k to be simply divided by *k* to obtain per nucleotide, assuming no overlap of variants. This method is more interesting for tetraploid cases with two different coalescent models for auto- and allo- tetraploid species respectively.

A different way to look at the difference of the three methods is “what heterozygosity would be predicted given the same relative size of the 1n *k*-mer peak”. In this comparison, we can easily see that the GenomeScope estimate will always generate higher heterozygosity estimates compared to the simple model, but smaller than Tetmer (**see the Figure underneath**). While looking at the plot, note that Tetmer does not use the same type of estimate, which makes the comparison somewhat unbalanced.

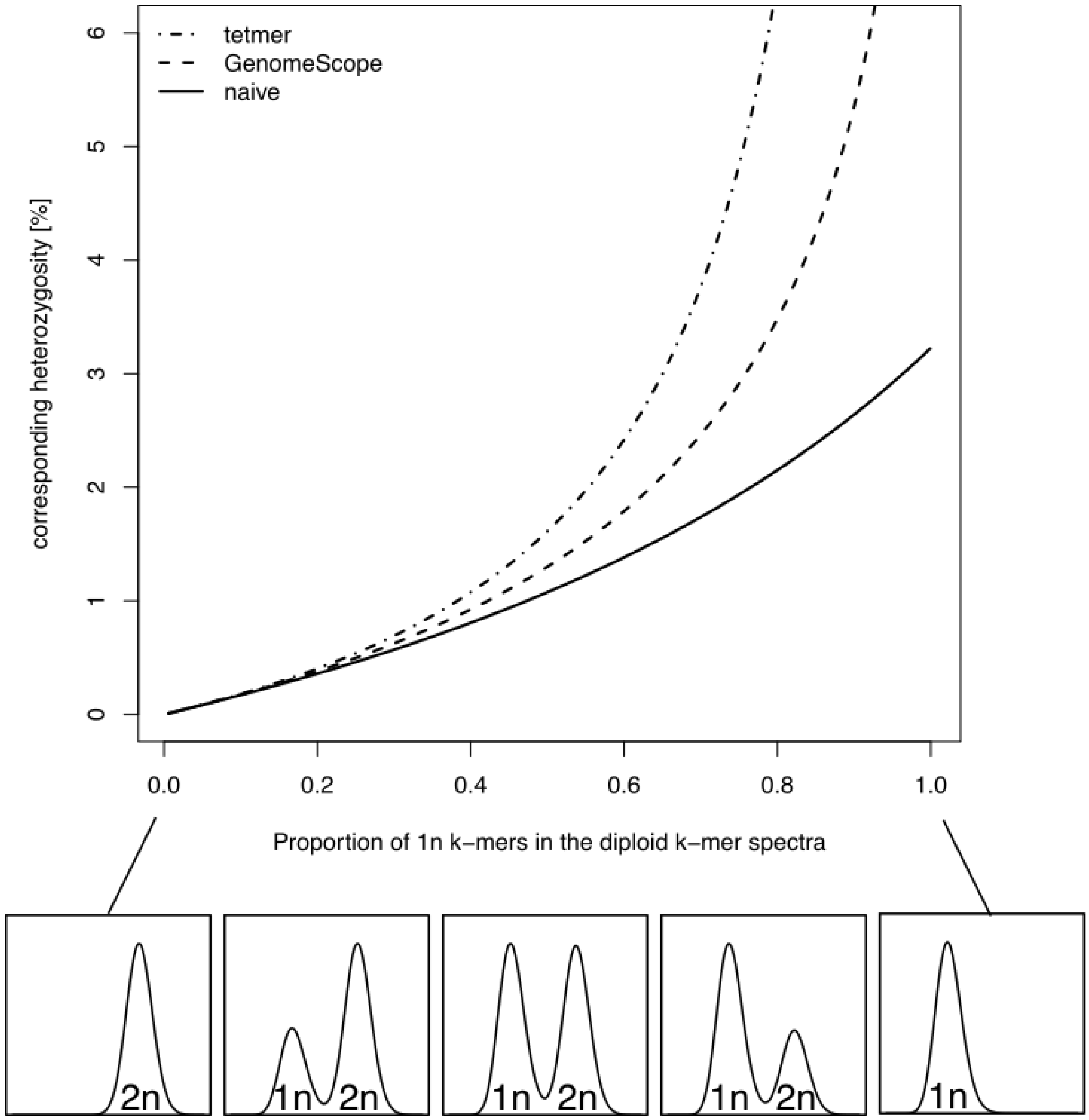

Sizes of peaks corresponding to heterozygosity estimates by different methods. The three available methods estimating heterozygosity differ in the interpretation of the relative size of the 1n peak. In the case of a small 1n peak (<0.2 of all the *k*-mers), all methods estimate similar levels of heterozygosity. With an increasing proportion of *k*-mers in the 1n peak, the estimate starts to differ. Notably, Tetmer does not estimate heterozygosity, but genetic diversity, which is not exactly the same measure.

Supplemental References

Altschul SF, Gish W, Miller W, Myers EW, Lipman DJ. 1990. Basic local alignment search tool. *J Mol Biol* **215**: 403–410.

Becher H, Brown MR, Powell G, Metherell C, Riddiford NJ, Twyford AD. 2020. Maintenance of Species Differences in Closely Related Tetraploid Parasitic Euphrasia (Orobanchaceae) on an Isolated Island. *Plant Communications* **1**: 100105.

Burkhardt S, Crauser A, Ferragina P, Lenhof H-P, Rivals E, Vingron M. 1999. *q* -gram based database searching using a suffix array (QUASAR). In *Proceedings of the third annual international conference on Computational molecular biology*, ACM, New York, NY, USA https://dl.acm.org/doi/10.1145/299432.299460.

Chee M, Yang R, Hubbell E, Berno A, Huang XC, Stern D, Winkler J, Lockhart DJ, Morris MS, Fodor SP. 1996. Accessing genetic information with high-density DNA arrays. *Science* **274**: 610–614.

Darwin Tree of Life Project Consortium. 2022. Sequence locally, think globally: The Darwin Tree of Life Project. *Proc Natl Acad Sci U S A* **119**: e2115642118.

Delcher AL, Kasif S, Fleischmann RD, Peterson J, White O, Salzberg SL. 1999. Alignment of whole genomes. *Nucleic Acids Res* **27**: 2369–2376.

Drmanac R, Labat I, Brukner I, Crkvenjakov R. 1989. Sequencing of megabase plus DNA by hybridization: theory of the method. *Genomics* **4**: 114–128.

Drmanac R, Labat I, Crkvenjakov R. 1991. An algorithm for the DNA sequence generation from k-tuple word contents of the minimal number of random fragments. *J Biomol Struct Dyn* **8**: 1085–1102.

Gutekunst J, Andriantsoa R, Falckenhayn C, Hanna K, Stein W, Rasamy J, Lyko F. 2018. Clonal genome evolution and rapid invasive spread of the marbled crayfish. *Nat Ecol Evol* **2**: 567–573.

Hänfling B, Nunn AD, Moccetti P, Wellcome Sanger Institute Tree of Life programme, Wellcome Sanger Institute Scientific Operations: DNA Pipelines collective, Tree of Life Core Informatics collective, Darwin Tree of Life Consortium. 2023a. The genome sequence of the stone loach, Barbatula barbatula (Linnaeus, 1758). *Wellcome Open Res* **8**: 518.

Hänfling B, Smith A, Wellcome Sanger Institute Tree of Life programme, Wellcome Sanger Institute Scientific Operations: DNA Pipelines collective, Tree of Life Core Informatics collective, Darwin Tree of Life Consortium. 2023b. The genome sequence of the nine-spined stickleback, Pungitius pungitius (Linnaeus, 1758). *Wellcome Open Res* **8**: 555.

Hirakawa H, Shirasawa K, Kosugi S, Tashiro K, Nakayama S, Yamada M, Kohara M, Watanabe A, Kishida Y, Fujishiro T, et al. 2014. Dissection of the octoploid strawberry genome by deep sequencing of the genomes of Fragaria species. *DNA Res* **21**: 169–181.

Idury RM, Waterman MS. 1995. A new algorithm for DNA sequence assembly. *J Comput Biol* **2**: 291–306.

Lander ES, Waterman MS. 1988. Genomic mapping by fingerprinting random clones: a

mathematical analysis. *Genomics* **2**: 231–239.

Lipman DJ, Pearson WR. 1985. Rapid and sensitive protein similarity searches. *Science* **227**: 1435–1441.

Lippert RA, Huang H, Waterman MS. 2002. Distributional regimes for the number of k-word matches between two random sequences. *Proc Natl Acad Sci U S A* **99**: 13980–13989.

Liu D, Singh GB. 2000. Profile based methods for genomic sequence retrieval. *SIGBIO Newsl* **20**: 6–13.

Li X, Waterman MS. 2003. Estimating the repeat structure and length of DNA sequences using L-tuples. *Genome Res* **13**: 1916–1922.

Mandeles S. 1968. Location of unique sequences in tobacco mosaic virus ribonucleic acid. *J Biol Chem* **243**: 3671–3674.

Marçais G, Kingsford C. 2011. A fast, lock-free approach for efficient parallel counting of occurrences of k-mers. *Bioinformatics* **27**: 764–770.

Mullikin JC, Ning Z. 2003. The phusion assembler. *Genome Res* **13**: 81–90.

Nurk S, Koren S, Rhie A, Rautiainen M, Bzikadze AV, Mikheenko A, Vollger MR, Altemose N, Uralsky L, Gershman A, et al. 2022. The complete sequence of a human genome. *Science*. https://www.science.org/doi/10.1126/science.abj6987 (Accessed June 27, 2023).

Nurk S, Walenz BP, Rhie A, Vollger MR, Logsdon GA, Grothe R, Miga KH, Eichler EE, Phillippy AM, Koren S. 2020. HiCanu: accurate assembly of segmental duplications, satellites, and allelic variants from high-fidelity long reads. *Genome Res* **30**: 1291–1305.

Ranallo-Benavidez TR, Jaron KS, Schatz MC. 2020. GenomeScope 2.0 and Smudgeplot for reference-free profiling of polyploid genomes. *Nat Commun* **11**: 1432.

Reinert G, Schbath S, Waterman MS. 2000. Probabilistic and statistical properties of words: an overview. *J Comput Biol* **7**: 1–46.

Shannon CE. 1948. A mathematical theory of communication. *Bell Syst Tech J* **27**: 379–423.

Sims D, Sudbery I, Ilott NE, Heger A, Ponting CP. 2014. Sequencing depth and coverage: key considerations in genomic analyses. *Nat Rev Genet* **15**: 121–132.

Smith NMA, Wade C, Allsopp MH, Harpur BA, Zayed A, Rose SA, Engelstädter J, Chapman NC, Yagound B, Oldroyd BP. 2019. Strikingly high levels of heterozygosity despite 20 years of inbreeding in a clonal honey bee. *J Evol Biol* **32**: 144–152.

Vurture GW, Sedlazeck FJ, Nattestad M, Underwood CJ, Fang H, Gurtowski J, Schatz MC. 2017. GenomeScope: fast reference-free genome profiling from short reads. *Bioinformatics* **33**: 2202–2204.
